# Os(II)-Bridged Polyarginine Conjugates: The Additive Effects of Peptides in Promoting or Preventing Permeation in Cells and Multicellular Tumor Spheroids

**DOI:** 10.1021/acs.inorgchem.1c00769

**Published:** 2021-05-12

**Authors:** Karmel S. Gkika, Sara Noorani, Naomi Walsh, Tia E. Keyes

**Affiliations:** † School of Chemical Sciences, National Centre for Sensor Research, Dublin City University, Dublin 9, Ireland; ‡ School of Biotechnology, National Institute for Cellular Biotechnology, Dublin City University, Dublin 9, Ireland

## Abstract

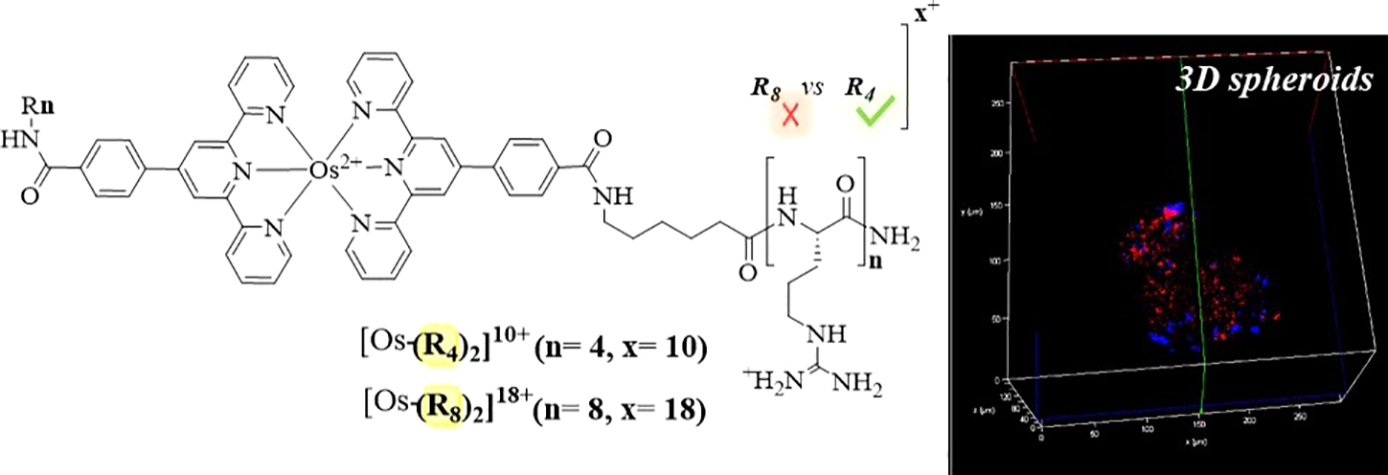

The preparation of two polyarginine conjugates of the complex Os(II) [bis-(4′-(4-carboxyphenyl)-2,2′:6′,2″-terpyridine)] [Os-(R_*n*_)_2_]^*x*+^ (*n* = 4 and 8; *x* = 10 and 18) is reported, to explore whether the R8 peptide sequence that promotes cell uptake requires a contiguous amino acid sequence for membrane permeation or if this can be accomplished in a linearly bridged structure with the additive effect of shorter peptide sequences. The conjugates exhibit NIR emission centered at 754 nm and essentially oxygen-insensitive emission with a lifetime of 89 ns in phosphate-buffered saline. The uptake, distribution, and cytotoxicity of the parent complex and peptide derivatives were compared in 2D cell monolayers and a three-dimensional (3D) multicellular tumor spheroid (MCTS) model. Whereas, the bis-octaarginine sequences were impermeable to cells and spheroids, and the bis-tetraarginine conjugate showed excellent cellular uptake and accumulation in two 2D monolayer cell lines and remarkable in-depth penetration of 3D MCTSs of pancreatic cancer cells. Overall, the data indicates that cell permeability can be promoted via non-contiguous sequences of arginine residues bridged across the metal centre.

## Abstract 2

In achiral Os(II) bisterpyridinyl complexes conjugated to polyarginine the peptide promoted cell permeability can be promoted via non-contiguous sequences of arginine residues bridged across the metal center.

## Introduction

Metal complex luminophores, most widely classified as phosphors, have emerged in the past decade as feasible alternatives to organic fluorophores for intracellular imaging and sensing.^1,2^ The attractive photophysical properties of such complexes have been widely reported, and for complexes of ruthenium, these include good photostability, long emission lifetimes, and Stokes-shifted emission in the red spectral region.^3−6^ While the emission maxima of complexes of Ir(III) and Ru(II) can be tuned toward the NIR, it can be synthetically challenging and such tuning may compromise photostability, exacerbated in the physiological conditions of temperature and buffered media, as well as emission quantum yield.^7−9^ Conversely, osmium (II) polypyridyl complexes exhibit similar advantages to Ru(II) for imaging but with the additional benefits of outstanding photostability and deep-red to NIR emission in the 700–850 nm spectral region, making them attractive candidates, in particular for tissue imaging (although still prone to the impact of the energy gap law). Although Os(II) polypyridyl complexes have to date, been much less explored for imaging applications than Ir or Ru, they are gaining increasing focus.^10−15^ Another advantage is that such complexes have long lifetimes in comparison to organic fluorophores. Os(II) complexes usually exhibit considerably shorter emission lifetimes than Ru(II) analogues and thus show low oxygen sensitivity. With lower oxidation potentials than ruthenium analogues, osmium complexes may also show sensitivity to other redox species without interference from oxygen. Typically molecular weight, charge, and lipophilicity mitigate against the membrane permeability of osmium (II) polypyridyl complexes, providing a barrier to in-cellulo applications but one rational approach to improve the efficacy of cellular uptake is to conjugate to short cationic peptides classified as cell-penetrating peptides (CPPs). The ability of cationic peptide sequences to cross the cell membrane and facilitate the uptake of small molecules was first demonstrated in 1965 by Ryser and Hancock with the cationic amino acid-mediated enhanced uptake of albumin, followed by studies on conjugation of poly-l-lysine to albumin and horseradish peroxidase.^16,17^ The most studied CPP is likely the arginine-rich HIV-Tat transduction protein (RKKRRQRRR) from immunodeficiency virus that has been widely shown to efficiently cross lipid bilayers.^18,19^ Indeed, homopolymers of arginine (polyarginines) have shown superior cellular uptake compared to other cationic analogues.^20^ The details of the mechanism by which oligoarginines permeate the membrane remain under investigation. The key pathway in live cells appears to be ATP-activated endocytosis,^21^ but there are also a number of studies that show that polyarginine can promote permeation through a passive mechanism,^22^ and they have been shown in artificial membranes to induce leakiness and topological changes at the membrane.^23^ Polyarginine interactions with cell surface lipids and formation of neutral complexes that transport across the bilayer have also been reported as well as surface attachment through interactions with heparan sulfate proteoglycans.^24−28^ Cargo transduction seems to occur for 6–11 Arg residues, with octaarginine (Arg8 or R8) and nona-arginine (Arg9) being most efficiently transported.^20^ We and others have reported that R5 or sequences of lower Arg residues are not CPPs,^29^ whereas R8 is very effective at promoting metal complex permeation.^30^

Barton and Brunner first reported the cellular uptake of cargo-carrying peptide rhodium complexes.^31^ Our group reported the efficient octaarginine-driven transport of an otherwise cell-impermeable Ru(II) polypyridyl compound, [Ru(bpy)_2_(pic)]^2+^, and its application in luminescence imaging.^30^ Puckett and Barton also reported the uptake of ruthenium (II)-dppz (dipyrido[3,2-a:2′,3′-c]phenazine) complexes conjugated to octaargine.^32^ Nona-arginine sequences containing phenylalanine residues have shown to enhance the cellular uptake of metallocene derivatives.^33^ Shorter polymers of arginine, below Arg6, are less efficient in cellular uptake, whereas longer polymers have shown unpredictable uptake and can even exert toxic effects.^20,30,34^ Sadler and co-workers reported improved uptake of a permeable Os(II) arene complex upon conjugation to R5 and R8 with the latter showing increased accumulation and toxicity.^35^ The monoarginine conjugate, however, showed a similar uptake to the unfunctionalized parent complex. Therefore, while there is a clear correlation between R-chain lengths and cellular uptake for metal complexes, the attachment of cationic amino acid residues below 5 or above 9 does not guarantee uptake in a predictable manner.

Our group exploited the use of non-specific and targeted CPPs to efficiently drive metal complexes across the cell membrane and target specific organelles.^9,36−40^ For example, the light-switching Ru^II^ complex with dppz conjugated to a nuclear localization signal sequence was highly effective in selectively driving the complex to the nucleus for imaging of chromosomal DNA using stimulated emission depletion (STED) microscopy,^39^ whereas the Ru-dppz conjugate bearing a mitochondrial targeting peptide localized at the mitochondria, confirmed by Raman spectroscopy and luminescence lifetime imaging.^41^ We recently demonstrated that polyarginines are equally effective in promoting the uptake of Os(II) polypyridyl complexes where R8 conjugated to [Os(bpy)_2_(pic)]^2+^ (bpy = 2,2-bipyridyl, pic = 2(4-carboxylphenyl) imidazo[4,5f]-[1,10]phenanthroline) facilitated the uptake of the highly photostable and NIR emitting complex in mammalian cell lines.^11^

While 2D monolayer studies can provide valuable information at the single cell level for a given cell line, three-dimensional (3D) cell models such as cell aggregates and spheroid structures provide a more physiologically relevant tumor tissue microenvironment.^42−44^ The multi-cellular layering of cells within spheroids leads to the formation of nutrient and oxygen gradients with hypoxic/necrotic regions toward the spheroid core and can better reflect permeation of a given species in vivo than in 2D cell culture where low cell density, lack of diffusion gradients, and cell–substrate interactions impact behavior.^45−47^ 3D cell models are of growing importance in drug discovery and toxicity testing. Their application requires luminescent probes that can deeply permeate the tumor spheroid and also probes capable of sensing within this environment. It has been shown that for fluorophores used for contrast, permeation of 2D models does not guarantee permeation of 3D models and that significant differences in the extent of permeation and destination of fluorescent probes can occur.^48^

Conjugation to polyarginines has been used to facilitate the uptake of gadolinium (III)-based contrast agents for magnetic resonance imaging.^49−51^ In addition, although polyarginines have been shown to be highly effective in promoting cellular uptake of transition metal luminophores, there have been no studies to date on whether this promotion extends to 3D cell models. The importance of tumor penetration is also crucial in the context of therapy by metallodrugs and photodynamic therapy agents as drug response in 3D cell models may differ when compared to 2D monolayers.^52−55^

A number of non-peptidic approaches to promote metal complexes in spheroid models have been reported recently. In the context of therapy, a dinuclear photo-oxidizing Ru^II^(TAP)_2_ (TAP = 1,4,5,8-tetraazaphenanthrene) complex was reported, showing in-depth photo-induced cell death of melanoma spheroids using two-photon excitation.^56^ Pt(II)–porphyrin probes have been employed for 2D and cell spheroid imaging particularly in the context of phosphorescence-based oxygen sensing.^57^ Haycock et al. reported the use of a small-molecule platinum (II) complex for oxygen mapping of melanoma spheroids using one-photon phosphorescence lifetime imaging microscopy (PLIM).^58^ More recently, two Ir(III) complexes were studied for *in vivo* PLIM O_2_ mapping.^59^ Papkovsky, Dmitriev, and co-workers have reported metalloluminophores with long emissive states ranging from Pt(II)–porphyrin probes to click-assembled Pd(II)–porphyrin nanoconjugates for NIR mapping of oxygen distribution in 3D microenvironments.^60,61^ Efficient cellular uptake of rather large porphyrins is often problematic in 2D monolayers and 3D models. While nanoparticles can provide a route for spontaneous or targeted cellular uptake, this strategy requires expertise in synthesis, and often cellular uptake is unpredictable. For example, despite the targeting capability of transferrin-conjugated gold nanoparticles, limited penetration was observed in multicellular spheroids.^62^

Herein, we investigate whether R8 is as effective in driving permeation of osmium NIR emitting luminophore in 3D tumor spheroids as it is in 2D cell monolayers. We recently reported the successful conjugation of two mitochondrial penetrating peptides (MPPs) to an achiral osmium (II) terpyridine complex.^40^ The conjugate showed relatively intense emission in the NIR and excellent photostability, making it suitable for tissue imaging. The advantage of this complex is that it does not form isomers and crucially it has conjugation points at the opposing apices of the complex, thus creating essentially a linear arrangement of the conjugation sites bridged by the metal. Here, the Os(II) parent complex [Os(tpybenzCOOH)_2_]^2+^ was conjugated to two polyarginine chains of varying lengths (R4 and R8), and we explore whether the optimal R8 requires a contiguous peptide structure for uptake or whether it can be accomplished in a bridged structure with a shorter peptide sequence. We report on the cellular uptake and localization in a cancerous and non-cancerous cell line using confocal and lifetime imaging. For the first time, we investigate the ability of the peptide conjugates to penetrate 3D cell models in pancreatic cancer multicellular tumor spheroids (MCTSs). To our knowledge, this is the first study of a polyarginine-driven osmium (II) conjugate used for 3D spheroid imaging.

## Experimental Section

### Materials

All chemicals and reagents, cell culture media, and corresponding components were purchased from Sigma-Aldrich (Ireland) and were used as received without further purification. Fetal bovine serum (FBS), heat-inactivated, was purchased from Biosciences Limited. Polyarginine sequences R8 and R4 (>95%) were procured from Celtek Peptides, TN, USA. Resazurin reagent was purchased from PromoKine, and co-localizing dyes were purchased from Thermo Fisher Scientific.

### Instrumentation

^1^H and COSY NMR spectra were recorded at 600 MHz using a Bruker spectrometer, and a deuterated solvent was used for homonuclear lock. The spectra were processed and calibrated against solvent peaks using Bruker Topspin software (v3.6.2). High-resolution mass spectrometry (HR-MS) was performed at the Mass Spectrometry Facility, NUI Maynooth. Analytical HPLC was performed on a Varian 940-LC with a photometric diode array (PDA) detector with peak monitoring at 280 and 490 nm channels. Gradient elution was applied using a 0.1% v/v TFA in the MeCN/water mixture. The mobile phase was of HPLC-grade quality and was filtered and purged with nitrogen prior to use. Prior to sample injection, the samples were filtered (0.8 μm pore size). The typical chromatographic run time was 20 min at a flow rate of 1 mL min^–1^.

### Synthesis

The [Os(tpybenzCOOH)_2_]^2+^ parent complex was prepared according to the synthetic procedure reported in the literature.^40,63^ The general procedure for the preparation of Os(II) bioconjugates is described below. The purity and characterization data are included in the Supporting Information.

#### Preparation of Os(II) Bioconjugates, [Os-(R_*n*_)_2_]^*x*+^ (*n* = 4 and 8; *x* = 10 and 18)

[Os(tpybenzCOOH)_2_]^2+^ (1 equiv), 20 mg of peptide (4 equiv), DIPEA (20 equiv), and PyBOP (8 equiv) were dissolved in 700 μL of DMF. The reaction mixture was stirred for 24 h at room temperature. The mixture was added dropwise to saturated NH_4_PF_6_ (aq) to obtain dark brown solids which were collected *via* vacuum filtration and washed with water and diethyl ether. The solids were dissolved in acetone and added dropwise to a TBAC/acetone solution to obtain the chloride salt of the peptide conjugate. The solids were washed with copious amounts of acetone and diethyl ether yielding [Os-(R_*n*_)_2_]^*x*+^ (*n* = 4 and 8; *x* = 10 and 18).

#### [Os-(R_8_)_2_]^18+^

^1^H NMR (600 MHz, MeOH-*d*_4_/D_2_O): δ (ppm) 9.31 (s, 4H), 8.86 (d, 4H), 8.44–8.35 (m, 8H), 7.85 (q, 4H), 7.39 (d, 4H), 7.19 (q, 4H), 5.22 (m, 9H), 4.44 (m, 18H), 3.88 (m, 3H), 3.42 (m, 9H), 3.22 (m, 9H), 3.10 (m, 3H), 2.96 (m, 2H), 2.82 (s, 1H), 2.18 (m, 2H), 1.99 (m, 7H), 1.70–1.09 (m, 76H), 0.96 (m, 2H), 0.82 (m, 9H). HR-MS(ESI-TOF) *m*/*z*: calcd for C_152_H_262_N_74_O_20_Os [M-bisR8^3+^ – Cl^–^] 1188.7296; found, 1187.7218.

#### [Os-(R_4_)_2_]^10+^

^1^H NMR (600 MHz, MeOH-*d*_4_/D_2_O): δ (ppm) 9.34 (s, 4H), 8.88 (d, 4H), 8.45–8.38 (m, 8H), 7.90 (q, 4H), 7.45 (d, 4H), 7.23 (q, 4H), 5.22 (m, 1 H), 4.49 (m, 10H), 3.93 (m, 5H), 3.75–3.3.65 (m, 7H), 3.38 (m, 1H), 3.27–2.99 (m, 6H), 2.27 (m, 1H), 2.03 (m, 2H), 1.67–1.05 (m, 36H), 0.91 (m, 1H), 0.85 (m, 6H). HR-MS(ESI-TOF) *m*/*z*: calcd for C_105_H_159_N_43_O_13_Os [M-bisR4^5+^ + 8H^+^ + Cl^–^] 493.0596; found, 493.1205.

### Photophysical Measurements

All absorbance measurements were performed on a Jasco V670 Spectrophotometer (Jasco Spectra Manager v2 software). Emission spectra were obtained using a Varian Cary Eclipse Fluorescence Spectrophotometer (Varian Cary Eclipse Software v1.1). The excitation and emission slit widths were set to 10 nm unless stated otherwise. Lifetime measurements were performed on a PicoQuant FluoTime 100 FLS TCSPC system using a 450 nm pulsed laser (PicoQuant PDL800-B) and an external Thurlby Thandar Instruments TGP110 10 MHz pulse generator. Luminescence lifetime data were acquired up to 10,000 counts, and decay curves were analyzed using PicoQuant Fluofit software and tail-fit statistical modeling (tail-fit criteria; 0.9 < χ^2^ < 1.1). The samples were de-aerated by solution-purging with nitrogen for 20 min. All lifetime measurements were performed in triplicate at room temperature (293 K) and are reported as mean ± SD.

### Monolayer Cell and 3D Spheroid Cell Cultures

Two cell lines were studied: an adherent mammalian cell line, Chinese hamster ovarian (CHO–K1), and a lung carcinoma cancer cell line (A549). Dulbecco’s modified Eagle’s medium (DMEM)/Hams F-12 was used for CHO cells and DMEM for A549 cells. Both media were supplemented with 10% FBS and 1% penicillin–streptomycin. The cells were grown at 37 °C with 5% CO_2_ and sub-cultured at 90% confluency.

The human pancreatic cancer cell line (HPAC) was cultured in Gibco Roswell Park Memorial Institute medium supplemented with 5% FBS at 37 °C with 5% CO_2_. HPAC cells were seeded at 2.5 × 10^3^ cells/well of 96-well round-bottom plates pre-coated with poly-Hema (poly-2-hydroxyethyl methacrylate, Sigma). Cells were allowed to be compact and form 3D spheroid structures after 72 h.

### Cytotoxicity Studies

The Alamar blue assay (Promocell GmbH) was used to assess the cell viability of A549 and CHO cells treated with the [Os-(R_4_)_2_]^10+^ probe. The cells were seeded in 96-well plates (flat-bottomed culture treated) at 10^4^ cells per well for 24 h at 37 °C with 5% CO_2_. The probe was added at concentrations 150, 100, 75, 50, 35, 25, 15, 10, 5, and 1 μM and incubated for 24 h prior to the addition of the Resazurin reagent (10% v/v) for 7 h at 37 °C in the absence of light. Absorbance readings were carried out at 570 and 600 nm (corrected for background subtraction) using a CLARIOstar (plus) (v 5.70) plate reader. The viability assay was performed in triplicate for each cell line.

### Confocal Laser Scanning Microscopy

Uptake studies were carried out for A549 and CHO cell lines. Cells were seeded at 1.5 × 10^5^ cells in 35 mm glass-bottom culture dishes (Ibidi, Germany). Cells were allowed to grow for 24 h at 37 °C with 5% CO_2_. The growth medium was removed, and a specific concentration of [Os-(R_*n*_)_2_]^*x*+^ (*n* = 4 and 8; *x* = 10^+^ and 18^+^) was added and allowed to incubate for 24 and 48 h at 37 °C with 5% CO_2_ in the dark. The dye/media solution was removed, and cells were washed twice with supplemented phosphate-buffered saline (PBS, 1.1 mM MgCl_2_ and 0.9 mM CaCl_2_). Cells were directly imaged using a Leica TSP DMi8 confocal microscope (63× oil immersion objective lens unless stated otherwise) with a heated stage at 37 °C.

[Os-(R_*n*_)_2_]^*x*+^ (*n* = 4 and 8; *x* = 10^+^ and 18^+^) was excited using a 490 nm white light laser, and the emission range was set to be between 650 and 800 nm. DRAQ7, a nuclear staining dye, was added (3 μM) to distinguish intact live cells from permeabilized/dead cells. The 633 nm laser was used to excite DRAQ7, and emission was collected between 635 and 750 nm. For co-localization studies, MitoTracker Deep Red (100 nM) and LysoTracker Green (50 nM) were used to selectively stain mitochondria and lysosomes, respectively. MitoTracker Deep Red was excited at 644 nm and emission was collected between 655 and 720 nm, and LysoTracker Green was excited at 504 nm and emission was collected at 511 nm.

### Phototoxicity

DRAQ7 was added to live A549 cells treated with [Os-(R_4_)_2_]^10+^ (30 μM/24 h). A group of cells were selected for continuous irradiation using 490 nm excitation (0.84 μW/cm^2^). Emission was collected between 650 and 800 nm for [Os-(R_4_)_2_]^10+^. The 633 nm laser was used to excite DRAQ7, and emission was collected between 635 and 750 nm. This was repeated for an untreated control sample exposed to continuous irradiation and stained with DRAQ7.

### Spheroid Treatment with Os-Compounds

Spheroids were treated with [Os-(R_4_)_2_]^10+^ at 30 μM for 24 h and 100 μM for 24 and 48 h. Control spheroids were treated with the parent complex [Os(tpybenzCOOH)_2_]^2+^ and [Os-(R_8_)_2_]^18+^ at 100 μM for 48 h. Non-treated spheroids were also prepared as a negative control. Spheroids were co-stained with DAPI at 10 μM for 90 min. Each spheroid was collected individually and embedded in collagen in eight-well chamber slides, incubated for 90 min, and observed under confocal microscopy.

### Preparation of Fixed Spheroid Sections

HPAC spheroids were treated with [Os-(R_4_)_2_]^10+^ at 100 μM for 48 h. The spheroids were collected, washed with PBS, and fixed in 4% NBF (neutral buffered formalin) for 30 min. The spheroids were then washed with PBS again and transferred into 10, 20, and 30% sucrose in PBS at 4 °C overnight until spheroids sank to the bottom. Spheroids were placed in an optimal cutting temperature (OCT) embedding matrix gel in a histology mold and placed at −80 °C overnight. Spheroids were cut into 5 μm sections using a cryostat. Sections were co-stained with DAPI. The slides were observed under confocal microscopy.

### Confocal Microscopy of HPAC Spheroids

For imaging, HPAC spheroids were plated in collagen-treated eight-well chamber slides. HPAC spheroids were directly imaged from the eight-well chamber using a Leica TSP DMi8 confocal microscope (10× dry and 40× oil immersion objective). *Z*-scanning was used to acquire images across the *z*-axis of the spheroid sample. On average, 45–50 images were acquired at approximately 5 μm apart. 3D Z-stack images were used for spheroid reconstructions and 2D projection images are also reported. The Os(II) parent complex and bioconjugates were excited using a 490 nm white light laser, and the emission range was set to be between 650 and 800 nm (a false red color was used for the Os(II) channel). DAPI was excited at 405 nm, and emission was collected between 423 and 580 nm.

### Evaluation of Spheroid Viability

The 3D spheroid viability was evaluated using Alamar Blue. Briefly, 2.5 × 10^3^ cells were seeded in each well of a 96-well round-bottom plate pre-coated with poly-Hema. The cells were allowed to grow and form spheroids for 72 h. The spheroids were then treated with [Os-(R_4_)_2_]^10+^ at 100 μM for 24 and 48 h. The Alamar Blue reagent at a concentration of 10% was added to each well and incubated for 5 h at 37 °C. Fluorescence was measured at 535/590 excitation/emission wavelengths on a plate reader (Biotek) using Gen4 software. Background fluorescence was measured by using a blank consisting of medium only. Percentage viability was calculated relative to untreated controls.

### Phosphorescence Lifetime Imaging Microscopy

Live A549 cells or HPAC spheroids were prepared and treated with [Os-(R_4_)_2_]^10+^ as described previously. Luminescence lifetime imaging was carried out using a PicoQuant 100 system attached to a Leica TSP inverted (DMi8) confocal microscope using a 63× or 40× oil immersion objective. Each sample was acquired for 120 s with a 512 × 512 resolution using the 405 nm. Data was analyzed using PicoQuant SymphoTime software.

## Results and Discussion

### Preparation and Characterization of the Bis-tetra and Bis-octa Arginine Os(II) Conjugates

**Figure 1:**
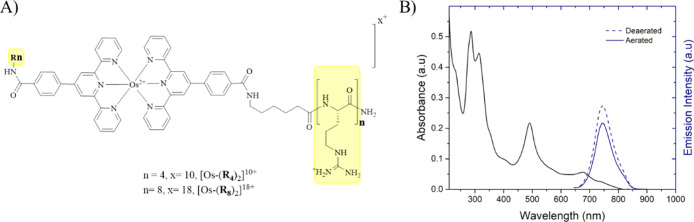
(A) General chemical structure of Os(II)-terpyridine-based polyarginine conjugates following amide coupling of R_*n*_ (*n* = 4 and 8) to [Os(tpybenzCOOH)_2_]^2+^ parent complex. (B) Absorbance and normalised emission spectra of [Os-(R_4_)_2_]^10+^ (30 μM/PBS buffer pH 7.4) under aerated and de-aerated conditions with λ_exc_ 490 nm and excitation and emission slit widths of 10 nm.

We reported recently on the preparation and photophysical characteristics of the achiral Os(II) parent complex, [Os(tpybenzCOOH)_2_]^2+^, used in this study.^40,64^ For an osmium polypyridyl complex, it exhibits a comparatively high emission quantum yield and in particular, as described, has the advantage from the perspective of the current application of a linear structure with bilateral conjugation sites, offering the possibility to extend a linear peptide sequence bridged by the metal center. The tridentate ligand structure also avoids the complexity of the possibility of preparation of isomers. Here, we explore the impact of polyarginine chains of different lengths to each carboxyl termini of the Os(II) complex, to understand in such an arrangement, if the octaarginine sequence we have observed to be so efficient in driving related metal complexes across the cell membrane and into the cytoplasm require that the arginine residues are contiguous and if longer arginine chains further improve the uptake. A comparative study on multivalent antimicrobial peptides reported enhanced antimicrobial activity for divalent metallocene conjugates.^65^ The general chemical structure of the conjugates, [Os-(R_*n*_)_2_]^*x*+^ (*n* = 4 and 8; *x* = 10^+^ and 18^+^), is shown in Figure 1A. Each polyarginine chain was composed of four or eight d-Arg residues. Studies have shown that internalization is not stereospecific as both d- and l-Arg enter cells efficiently.^20^

Bis-octaarginine and bis-tetraarginine conjugation to the carboxyl termini of [Os(tpybenzCOOH)_2_]^2+^ was achieved via amide coupling using PyBOP/DIPEA following the procedures reported previously.^40,41^ The structure and purity of the conjugates were confirmed by ^1^H and COSY NMR, reverse-phase HPLC (PDA 490 nm), and high-resolution LC mass spectrometry. Both polyarginine conjugates showed very similar photophysical properties in PBS (pH 7.4) to the parent complex and Os^II^ MPP previously reported.^40^ The absorbance and emission spectra of [Os-(R_4_)_2_]^10+^ (30 μM) are shown in Figure 1B. The emission maximum centered at 754 nm coincides well with the biological window and is usefully situated outside of the range of cellular autofluorescence. [Os-(R_4_)_2_]^10+^ and [Os-(R_8_)_2_]^18+^ in aerated PBS (pH 7.4) exhibited luminescence lifetimes of 89.6 ± 5.3 ns and 89.2 ± 6.1, respectively, which, as for their related MPP conjugates, were only modestly affected by de-aeration (τ_Os–R4_ 103 ± 6.9 ns and τ_Os–R8_ 108.6 ± 11.1 ns).

### Uptake Studies of [Os-(R_*n*_)_2_]^*x*+^ Conjugates

As reported previously, the parent complex without peptide is cell membrane impermeable. Uptake of the Os(II) bis-octaarginine conjugate was studied in live A549 lung carcinoma and a non-cancerous CHO cell line. Surprisingly, confocal laser scanning microscopy (CLSM) revealed that bis-conjugation to octaarginine did not facilitate transport of the complex.^66^ The conjugate remained as impermeable to the cell membrane as the parent Os(II) complex at 30 μM/24 h and 100 μM/24 h (Figure S12). As expected, in the absence of any permeation, A549 and CHO cells remained healthy as confirmed by co-staining with DRAQ7, a dye which stains the nucleus of damaged/dead cells.

As previously mentioned, evidence suggests that cargo cellular uptake is enhanced with an increase in arginine chain length for polyarginine CPPs. However, whereas it has been widely observed that R8 or R9 provides optimal uptake, the inhibition of uptake with longer polyarginine chains has also been noted. For example, Sugiura et al. noted, consistent with our data, that fluorescently labeled R8 was efficiently drawn across live cell membranes, whereas R16 was not.^67^ Studies have also shown that the cellular uptake ability of oligoarginine conjugates depends on the total number of Arg residues and not necessarily on the exact arginine sequence.^68,69^ As the chain length increases, the uptake behavior becomes less predictable. For example, studies found that R15 conjugation showed superior uptake to R20–R30 and R75 proved to be toxic toward cells.^20^ Long peptides of lysine have also shown cellular toxic effects.^70^ The reason for reduced uptake by longer arginine chains is not entirely clear as the mechanism of arginine-mediated uptake is still debated. If translocation is the key mechanism, it may be that the longer chain lengths associate too strongly with the membrane or do not cause the appropriate degree of curvature at the cell membrane or are an inappropriate length to span the membrane in endocytosis. It is notable that long-term incubation with the complex did not cause cytotoxicity, confirmed by the absence of DRAQ7 from the nucleus, indicating that membrane destabilization by these long R chains can be excluded.

As reported previously, R8 promotes the uptake of Ru and Os(II) polypyridyl complexes across live cell membranes, so we then conjugated two R4 chains to each terminus of the complex.

**Figure 2:**
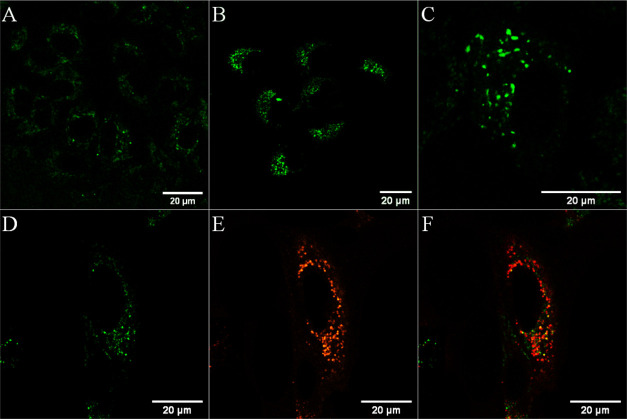
Uptake and co-localization studies of [Os-(R_4_)_2_]^10+^ in live A549 cells where the osmium channel is shown in green. Cells were incubated in the absence of light with 30 μM [Os-(R_4_)_2_]^10+^ for (A) 24 h and for (B,C) 48 h. Co-localization studies at 48 h with Lysotracker Green (50 nM) confirmed lysosomal confinement evident by the overlap of the (D) osmium channel with (E) Lysotracker Green (orange) in the (F) overlay image (Pearson’s coefficient = 0.69). A 490 nm white light laser was used to excite the conjugate, and emission was collected between 650 and 800 nm. The LysoTracker Green dye was excited at 504 nm and emission was collected at 511 nm.

In contrast to [Os-(R_8_)_2_]^18+^, [Os-(R_4_)_2_]^10+^ was rapidly and efficiently internalized into live A549 and CHO cells at 30 μM following 24 h incubation in the dark. As shown in Figure 2, emission from the cytoplasm was evident at 24 h and by 48 h as punctuate staining. Previously reported ruthenium octaarginine conjugates have generally been found to be nuclear excluding with non-specific distribution throughout the cytoplasm.^38,39^ Uptake of an osmium polypyridyl phenanthroline imidazole complex conjugated to R_8_, [Os(bpy)_2_(pic-arg_8_)]^10+^ showed comparable uptake to its ruthenium analogue, although unlike the Ru analogue, nuclear penetration of the osmium complex was observed in CHO cells particularly under photoirradiation.^11^ The increased lipophilic character of the osmium complex compared to its ruthenium analogue was suggested as a reason for its nuclear permeation.^11^ Given the punctate distribution of the complex at later time points, to establish distribution of [Os-(R_4_)_2_]^10+^, co-localization studies were carried out using Lysotracker Green for the lysosomes and MitoTracker Deep Red for the mitochondria. Staining with DRAQ7 was used to identify damaged cells. A Pearson’s coefficient value of 0.56 revealed only moderate co-localization with MitoTracker Deep Red at 30 μM/24 h, suggesting that at these conditions, the conjugate enters but is not exclusively localized at the mitochondria (Figure S13).

**Figure 3:**
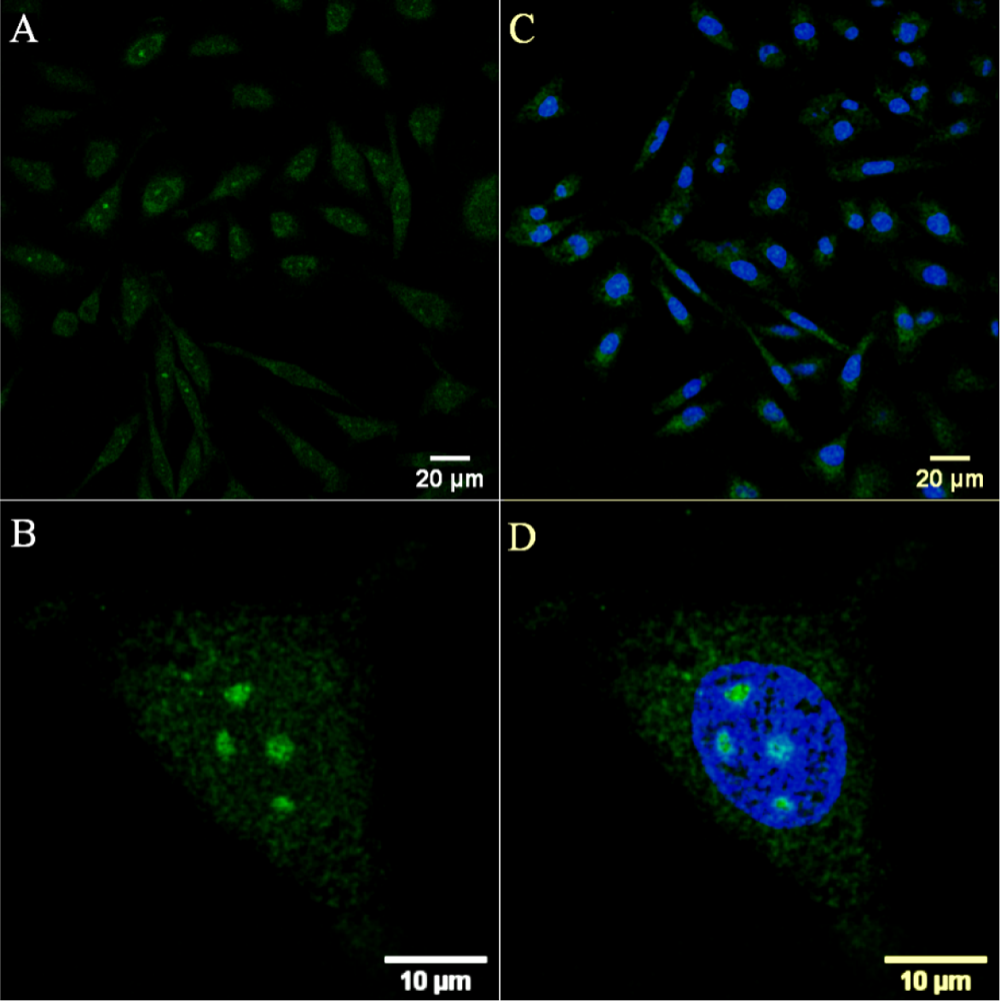
Confocal luminescence images of [Os-(R_4_)_2_]^10+^ in CHO cells. Live cells were incubated with 30 μM [Os-(R_4_)_2_]^10+^ for 24 h in the absence of light and co-stained with DRAQ7. (A,C) The distribution of the conjugate (in green) is shown in a group of cells co-stained with DRAQ7 (in blue). (B,D) Closed-up image of a single A549 cell shows nucleoli staining. The 633 nm laser was used to excite DRAQ 7 and emission was collected between 635 and 750 nm.

As shown in Figure 2D–F, the punctuate staining of [Os-(R_4_)_2_]^10+^ (green) at 30 μM/48 h co-localized well with Lysotracker Green (orange) confirming localization of the conjugate in lysosomal structures. Trafficking to the lysosomes of HIV-TAT and octaarginine following uptake has been reported previously and has been linked to an endocytic uptake mechanism.^71^ A similar uptake and distribution in A549 cells were observed when incubated for 24 h at higher complex concentrations of 100 μM [Os-(R_4_)_2_]^10+^ (Figure S14). Uptake of [Os-(R_4_)_2_]^10+^ was also studied in a non-cancerous CHO cell line. As shown in Figure 3A,B, CHO cells treated with [Os-(R_4_)_2_]^10+^ at 30 μM and incubated for 24 h showed extensive cytoplasmic and nucleoli staining. In this case, nuclear penetration did not concern a photo- or thermally activated process as penetration was observed following incubation of [Os-(R_4_)_2_]^10+^ in the dark.

The [Os-(R_4_)_2_]^10+^ conjugate exhibited temperature-dependent uptake as cell permeation of A549 and CHO cells was inhibited at 4 °C, suggesting that an activated process, such as endocytosis, is involved for cellular entry (Figure S15). Previously reported octaarginine conjugates of metal complex cargo have been shown to be internalized *via* an activated membrane transport process.^11,72,73^

Co-staining studies using DRAQ7 confirmed that the A549 cells remained viable after both 24 h and 48 h treatment with the conjugate at 30 μM (Figure S16). The absence of DRAQ7 from the cell nuclei of A549 cells treated with the conjugate at a higher concentration of 100 μM/24 h confirmed that no cell death was induced at this higher concentration (Figure S14A,B). After extending the incubation time for 100 μM [Os-(R_4_)_2_]^10+^ to 48 h, some DRAQ7 staining was evident, indicating modest cytotoxic effects at extended incubations and higher probe concentration (Figure S14C–E). In order to assess potential photo-induced toxic effects, a group of A549 cells, pretreated with [Os-(R_4_)_2_]^10+^ (30 μM/24 h) and stained with DRAQ7, was continuously irradiated using 490 nm excitation (at 0.84 μW/cm^2^, approximately 3 times higher than the imaging intensity). Under these conditions, no DRAQ7 was found to enter cell nuclei following 3 h of irradiation, confirming that the osmium conjugate is non-photocytotoxic under these conditions (Figure S17). We attribute this to the insensitivity of the excited state of this species to molecular oxygen. It is not quenched by O_2_ and thus does not generate cytotoxic singlet oxygen under irradiation, and the low excited-state oxidation potential of the complex limits photoredox reactions with DNA or protein constituents. The extent of nucleoli staining of [Os-(R_4_)_2_]^10+^ and cellular damage in CHO cells, following 24 h of incubation at 30 μM, was evident by the entry of DRAQ7 (blue) in several cell nuclei (Figure 3C,D).

The viability of A549 and CHO cells treated with [Os-(R_4_)_2_]^10+^ was assessed by incubating the conjugate overnight at concentrations up to 150 μM at 37 °C with 5% v/v CO_2_ using the Alamar Blue assay (Figure S18). The reduction of the resazurin reagent to resorufin was used to directly measure cell viability. The conjugate was found to be remarkably non-toxic toward A549 cells with an IC_50_ exceeding 150 μM and cell viability was above 71% up to 100 μM. [Os-(R_4_)_2_]^10+^ was found to be somewhat more toxic to CHO cells where viability decreased to <70% above 35 μm with an IC_50_ ≈ 75.1 ± 1.1 μM. It is likely that the cytotoxicity in CHO cells is the result of wider distribution of the conjugate in this cell line, which is evident by confocal imaging where cytoplasmic staining and nucleoli staining were observed. The previously reported [Os(tpybenzCOOH)_2_]^2+^ complex conjugated to two MPPs exhibited a concentration-dependent localization and cytotoxicity toward HeLa and MCF 7 cells with an IC_50_ ≈ 30 μM.^40^ However, here conjugation of the Os(II) complex to two tetraarginine chains leads to significantly decreased toxicity likely owing to the preferential cytoplasmic staining of the conjugate. Overall, [Os-(R_4_)_2_]^10+^ is not photocytotoxic, and the dark toxicity of the bis-tetraarginine Os(II) conjugate toward A549 and CHO cell lines is low and comparable to the reported ruthenium and osmium octaarginine compounds.^11,37^ In addition, [Os-(R_4_)_2_]^10+^ was less toxic than the octaarginine conjugate [Ir(dfpp)_2_(picCHONH) (Arg_8_]^9+^ with IC_50_ values of 54.1 and 35 μM against CHO and SP2 cells, respectively.^73^

### PLIM and Cell Mapping

The relatively long emission lifetimes of the metal complexes render them potentially useful as probes for mapping of the environment of live cells using luminescence lifetime imaging. For example, Ir(III) and Ru(II) polypyridyl complexes have been employed as O_2_ sensors using PLIM studies.^29,52,53^ PLIM was carried out here to investigate the emission lifetime of the conjugate in the environment of live cells.

**Figure 4:**
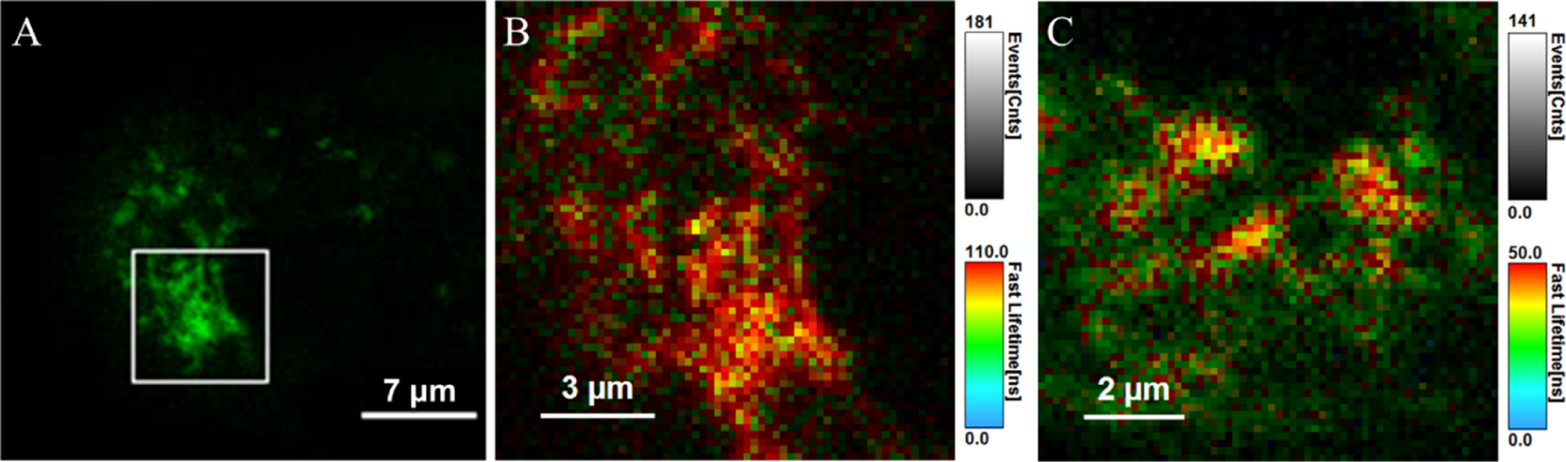
Luminescence lifetime imaging [Os-(R_4_)_2_]^10+^ at 30 μM in live A549 cells. (A) Confocal image of a single cell following conjugate uptake at 24 h and (B) lifetime distribution in the expanded cytoplasmic region of the cell. (C) PLIM acquired following uptake at 48 h. The PLIM images were acquired using the 405 nm excitation laser line. The PLIM images of the entire cell of (B) and (C) and corresponding emission decays are shown in the Supporting Information (Figures S19–S20).

Figure 4 shows the false-color images of the lifetime distributions of the Os(II) probe in A549 cells at 30 μM following uptake at 24 and 48 h incubation. The emission lifetime of the conjugate was found to vary with localization, attributed to environmental effects. When taken up in the cytoplasm and partially confined in mitochondria at 24 h, the lifetimes fit to a tri-exponential decay with a very short component (τ_3_) that was present under all circumstances of 2 ns and attributed to background scatter/reflectance. The dominant amplitude component (67%) of the decay, τ_1_, was measured as 92.2 ns ± 2.9 ns with a second component of lifetime, τ_2_, recorded as 15.8 ± 1.5 ns (25%). Notably, when confined to the lysosomes and the surrounding cytoplasm at 30 μM/48 h, the [Os-(R_4_)_2_]^10+^ conjugate exhibited significantly reduced lifetimes of 37 ± 1.8 ns (54%) and 9.3 ± 0.6 ns (32%). Although O_2_-insensitive, the conjugate is expected to respond to redox-active species in the sub-cellular environment, but response on the basis of the pH of the lysosome can be excluded as the emission lifetime of the parent complex in aqueous solution (pH 4.1) showed no response to acidic pH (Figure S10). The oxidation potential of osmium is typically 300 mV lower than comparable Ru analogues, and for the parent complex here, the oxidation potential is 656 mV versus Ag/AgCl.^40^ The lysosome is an acidic organelle with a rich cocktail of redox-active species including metalloenzymes and thiols likely to quench and thus modulate the excited-state lifetime of this complex. The absence of O_2_ sensitivity but potential redox sensitivity to other species offers an advantage of Os polypyridyl complexes over ruthenium and iridium complexes in imaging, offering opportunities to monitor localization and metabolic changes in cells or tissues without interference from oxygen.

### Confocal Imaging of HPAC Spheroids

The excellent membrane permeability of the bis-tetraarginine Os(II) conjugate, its low photocytotoxicity, and NIR emission suggest its suitability as a probe for tissue imaging, and to date, there have been no applications of Os luminophores applied as 3D multicellular spheroids imaging probes. However, as noted, multicellular spheroids present a very different microenvironment. Thus, we investigated their penetration into 3D MCTSs. 3D cell spheroids are prepared by culturing cancer cells in a non-adherent environment, which promotes the formation of aggregates of cell layers.^42−45^

We performed studies on MCTSs composed of HPACs grown in ultra-low attachment well plates for 3 days. The spheroids were incubated with the [Os-(R_4_)]^10+^conjugate at 30 and 100 μM for 24 and 48 h each. For comparison, the ability of the Os(II) parent complex and the bis-octaarginine conjugate, [Os-(R_8_)_2_]^18+^, to penetrate 3D spheroids was also assessed by incubating with the compounds at 100 μM for 48 h. In the latter cases, no spheroid penetration or accumulation was observed for either parent or bis-octaarginine compound, consistent with studies on 2D cell monolayers (Figure S21).

**Figure 5:**
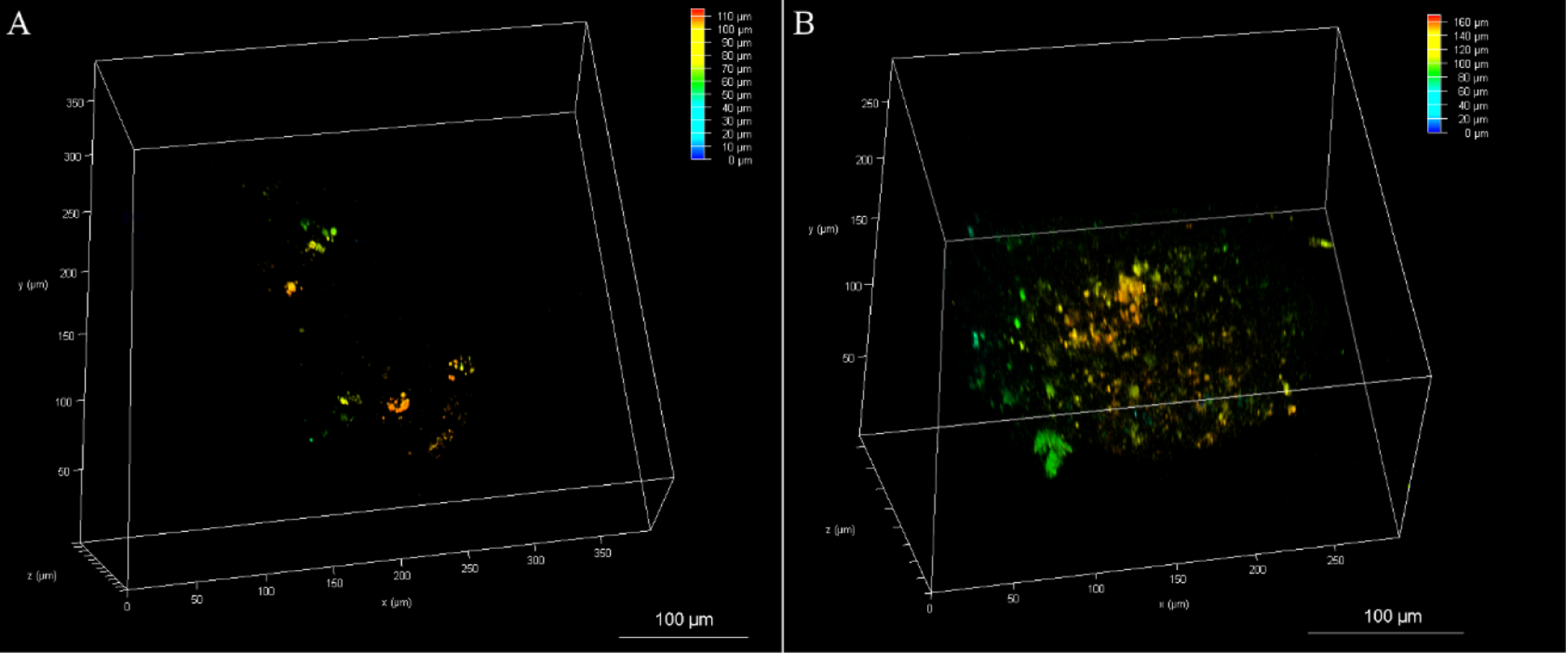
3D reconstruction depth coding images of whole live HPAC spheroids treated with [Os-(R_4_)_2_]^10+^ at (A) 30 μM/24 h and (B) 100 μM/24 h. Confocal images were acquired at different planes in the *z* direction throughout the spheroids (from the bottom to above each spheroid). A 490 nm white light laser was used to excite the conjugate, and emission was collected between 650 and 800 nm. Scale bar reads 100 μm.

Conversely, confocal luminescence microscopy studies revealed that the bis-tetraarginine conjugate readily permeates the HPAC spheroids. It is important to note that the probes are incubated with the live spheroid in aqueous buffer/media without application of a permeant such as detergent or solvent. As shown in the 3D reconstruction depth coding images, spheroid penetration was evident following incubation with [Os-(R_4_)]^10+^ at 30 μM/24 h, although staining at the center of the spheroid, at 72 ± 5 μm, was not observed at this concentration (Figure 5A). However, by increasing the complex concentration to 100 μM/24 h, extensive probe penetration and distribution to the core of the spheroid were achieved (Figure 5B). Hambley et al. reported on the depth of penetration of a series of platinum complexes in cancer cell spheroids, observing that it was inversely proportional to the rate of cellular uptake.^54^ It is likely that rapid accumulation of the probe at 30 μM results in penetration and accumulation of complex at the first few cell layers, leaving insufficient concentration of probe to measurably diffuse to the center of the MCTS at lower concentrations. Full Z-stack images are shown in the Supporting Information (Figures S22–S23).

**Figure 6:**
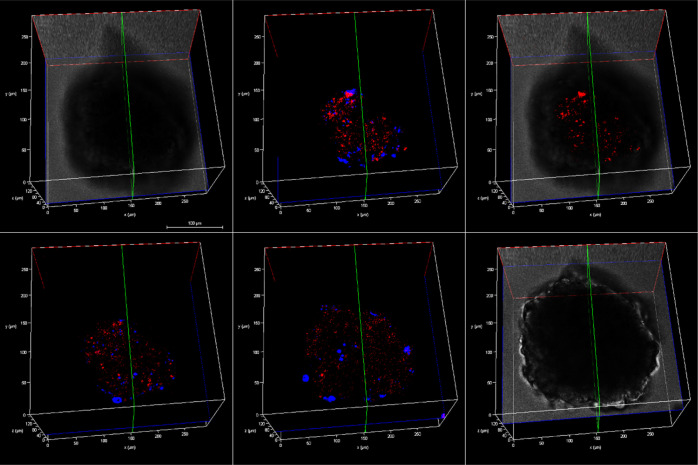
Z-stack images of a single live HPAC spheroid pre-treated with [Os-(R_4_)_2_]^10+^ (100 μM/48 h) and co-stained with DAPI (10 μM). Each image corresponds to the cross section from the bottom to the upper part along the *z*-axis. Representative cross sections are shown using bright-field contrast as the background. Scale bar reads 100 μm. A 490 nm white light laser was used to excite the conjugate, and emission was collected between 650 and 800 nm. The 405 nm excitation laser was used to excite DAPI, and emission was collected between 423 and 580 nm.

**Figure 7:**
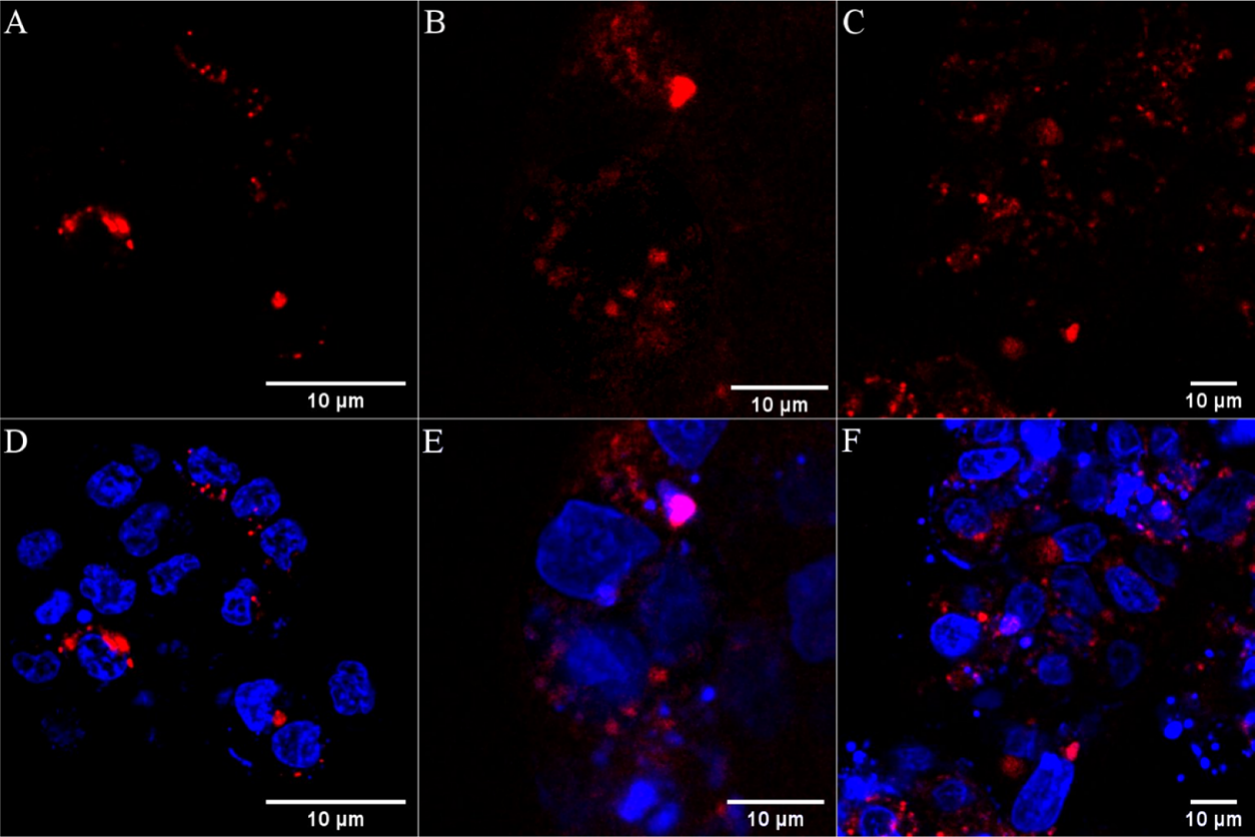
Confocal images (2D projection) of HPAC spheroid regions treated with Os-(R_4_)_2_ at (A) 30 μM/24 h, (B) 100 μM/24 h, and (C) 100 μM/48 h at 37 °C. The spheroids were co-stained with DAPI (10 μM) and (D–F) overlay images with DAPI channel. A 490 nm white light laser was used to excite the conjugate, and emission was collected between 650 and 800 nm. The 405 nm excitation laser was used to excite DAPI, and emission was collected between 423 and 580 nm (40× obj.).

Figure 6 shows representative z-stack images of a single live HPAC spheroid treated with [Os-(R_4_)_2_]^10+^ at 100 μM for 48 h. Emission from the conjugate (in red) is observed throughout cell layers and deep into the spheroid core. A nuclear localizing dye, DAPI (in blue), was used as a co-staining contrast dye. Figure 7 shows the extent of probe distribution in spheroid regions following incubation with [Os-(R_4_)_2_]^10+^, where uniform cytoplasmic staining, that is mainly nuclear excluding, is observed at 100 μM/24 h and 48 h.

Furthermore, following treatment with the tetraarginine conjugate, the live spheroids were examined for morphological changes by comparing with non-treated (control) spheroids, and the viability assay showed no evidence of toxicity (Figure S24).

**Figure 8:**
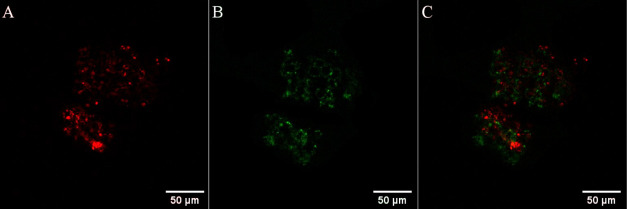
Confocal images of a single live HPAC spheroid treated with [Os-(R_4_)_2_]^10+^ (100 μM/48 h). A 490 nm white light laser was used for excitation, and emission was collected between (A) 650 and 800 nm; Os(II) channel and (B) 500–570 nm; autofluorescence window. (C) Os(II)/autofluorescence channel overlay.

A key advantage of Os(II) polypyridyl complexes is that they exhibit NIR emission well resolved from the autofluorescence window. Emission from cellular fluorophores, such as nicotinamide adenine dinucleotide (NAD^+^/NADH) and flavins, occurs mainly at shorter light wavelengths (350–550 nm); thus; in detecting fluorophores emitting below 700 nm, autofluorescence can be readily filtered out.^4,74,75^ [Os-(R_4_)_2_]^10+^ exhibits an emission maximum centered at 754 nm, and the emission collected here between 650 and 800 nm corresponding to the Os(II) channel (Figure 8A) avoids any background signal from biological autofluorescence (Figure 8B). Furthermore, as shown in the above PLIM images, the relatively long lifetimes of these probes also enable facile discrimination of contributions from autofluorescence in lifetime data.

To further evaluate the distribution of the conjugate inside the spheroids, fixed cryosections of HPAC spheroids that were pre-treated with [Os-(R_4_)_2_]^10+^ (100 μM) when live were prepared and imaged. The confocal fluorescence images confirm the wide distribution of the conjugate throughout the spheroid (Figures S25–S26). Punctuate staining and overlap with DAPI dye in the fixed samples suggest preferential accumulation of the conjugate at certain regions and some nuclear penetration and localization. The latter was not noted in the live pancreatic spheroid samples. However, it is important to note that the process of fixation has a profound impact on the cellular structures and can alter the distribution of probes within cells. Indeed, redistribution of nona-arginine-modified fluorescent dyes was noted after fixation where nucleolus migration was observed.^76^ Furthermore, as shown in the PLIM image of punctuate staining of [Os-(R_4_)_2_]^10+^ in fixed HPAC spheroids (Figure S27), the lifetime distribution is very uniform. Here, the decay conforms to a bi-exponential fit where the osmium conjugate shows only a single-component decay with a lifetime of 16.4 ns, which is short-lived than in solution (τ ≈ 89.6 ± 5.3 ns) but comparable to the lifetimes recorded in the 2D monolayer cell studies. However the lifetime of [Os-(R_4_)_2_]^10+^ and its uniformity are likely to reflect the sample fixation, which causes extensive cross-linking of protein structures into a gel state within the cell.^77,78^

## Conclusions

Using an achiral Os(II) bisterpyridinyl-coordinated complex with linear bilateral conjugation sites, we demonstrate that assembly of polyarginine at opposing ends of the structure exhibits an additive effect in terms of cargo cellular permeation. Whereas the parent complex is completely impermeable to live 2D and 3D cells, the octaarginine conjugate prepared as two tetraarginine moieties conjugated to the termini of the Os center shows excellent permeability and behaves in an analogous way to previously reported octaarginine derivatives of related complexes. In contrast, when two octaarginine peptides are appended to the termini, the conjugate is rendered completely impermeable to the 2D and 3D cell cultures.

The [Os-(R_4_)_2_]^10+^ conjugate exhibited exceptionally low cytotoxicity and photocytotoxicity and an emission maximum at 754 nm coinciding with the biological window. The [Os-(R_4_)_2_]^10+^ emission is essentially oxygen-insensitive but exhibits a relatively long-lived emission lifetime of approximately 89 ns in PBS that facilitates its use in phosphorescence lifetime imaging. Uptake was found to be efficient in all cell lines explored, but the distribution varied with cell type and where [Os-(R_4_)_2_]^10+^ accessed the lysosome its emission lifetime was significantly reduced likely attributed to emission quenching redox-active species in that organelle. We examined the application of conjugates in imaging a multilayer pancreatic cancer cell model; to our knowledge, this is the first example of an Os luminophore applied to imaging of 3D multicellular spheroids. While the parent complex and [Os-(R_8_)_2_]^18+^ conjugate were found to be impermeable, widespread and in-depth staining into the 3D MCTS was achieved with [Os-(R_4_)_2_]^10+^ without any impact on cellular function. The emission collected was well outside the autofluorescence window toward the NIR region of the spectrum suitable for tissue imaging. Our data demonstrate that the efficiency of octaarginine as a permeant does not require contiguous arrangement of the amino acids but shows that the sequence can be bridged at the luminophore termini. This approach may be more broadly applicable and could pave the way to novel compounds that combine shorter peptide sequences such as signal and CPP sequences and could also address issues in peptide synthesis concerning cumulative lower yields with longer peptides. This work also illustrates the value of non-cytotoxic osmium derivatives as probes for studying the MCTS environment.

## Supporting Information Available

NMR, mass spectrometry, and RP-HPLC data; photophysical data; confocal and lifetime images of 2D cell monolayers; and cytotoxicity assays and imaging of 3D multicellular pancreatic spheroids (PDF)
