# Supplementary material for: Os(II)-Bridged Polyarginine Conjugates: The Additive Effects of Peptides in Promoting or Preventing Permeation in Cells and Multicellular Tumor Spheroids
Source: Inorg Chem. 2021 May 12;60(11):8123–34. doi: 10.1021/acs.inorgchem.1c00769 (PMC8277133; doi:10.1021/acs.inorgchem.1c00769)
Supplement: Supplementary file 1 — ic1c00769_si_001.pdf [file ic1c00769_si_001.pdf]

Supporting Information

Os(II) Bridged Polyarginine Conjugates; The Additive effects of Peptides in Promoting or Preventing Permeation in Cells and Multicellular Tumor Spheroids.

Karmel S. Gkika[a], Sara Noorani[b], Naomi Walsh[b], Tia E. Keyes[a*].

[a]School of Chemical Sciences, National Centre for Sensor Research, Dublin City University, Dublin 9, Ireland.
[b]School of Biotechnology, National Institute for Cellular Biotechnology, Dublin City University, Dublin 9, Ireland.

Contents

NMR Analysis S-2

Mass Spectrometry S-4

RP-HPLC Analysis S-5

Photophysical studies S-6

Cell studies S-9

Confocal images 2D cell monolayers S-9

Cytotoxicity assay S-13

Luminescence Lifetime Imaging S-14

3D Multicellular pancreatic spheroids S-16

NMR Analysis

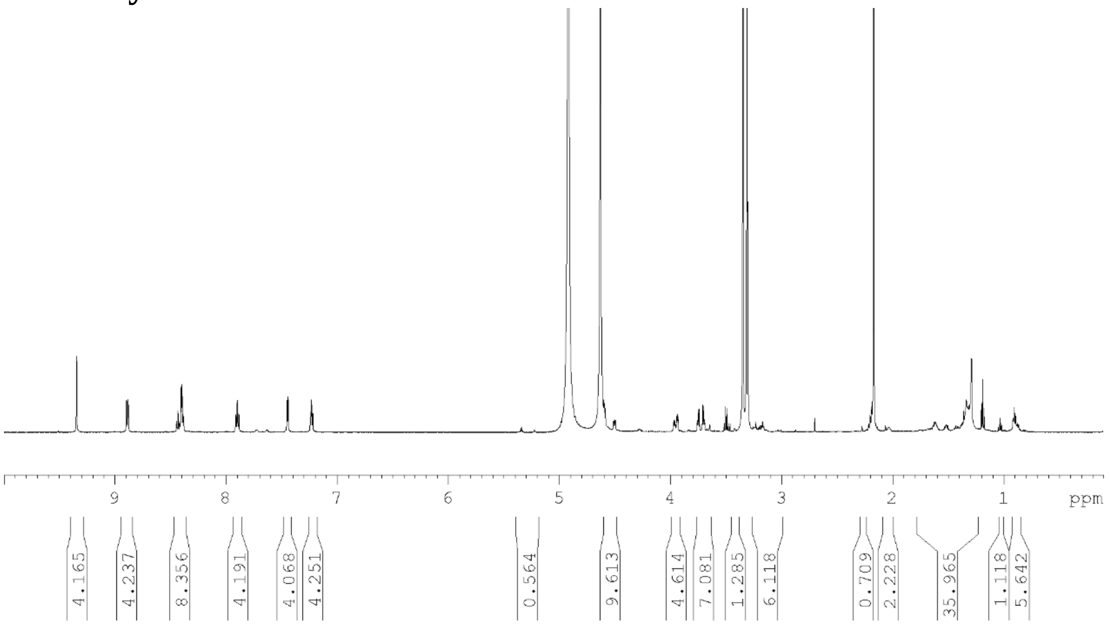

Figure S 1: ^{1}H NMR analysis of $[Os\text{-}(R_4)_2]^{10+}$ conjugate (MeOH-d_4/D_2O, 600 MHz)

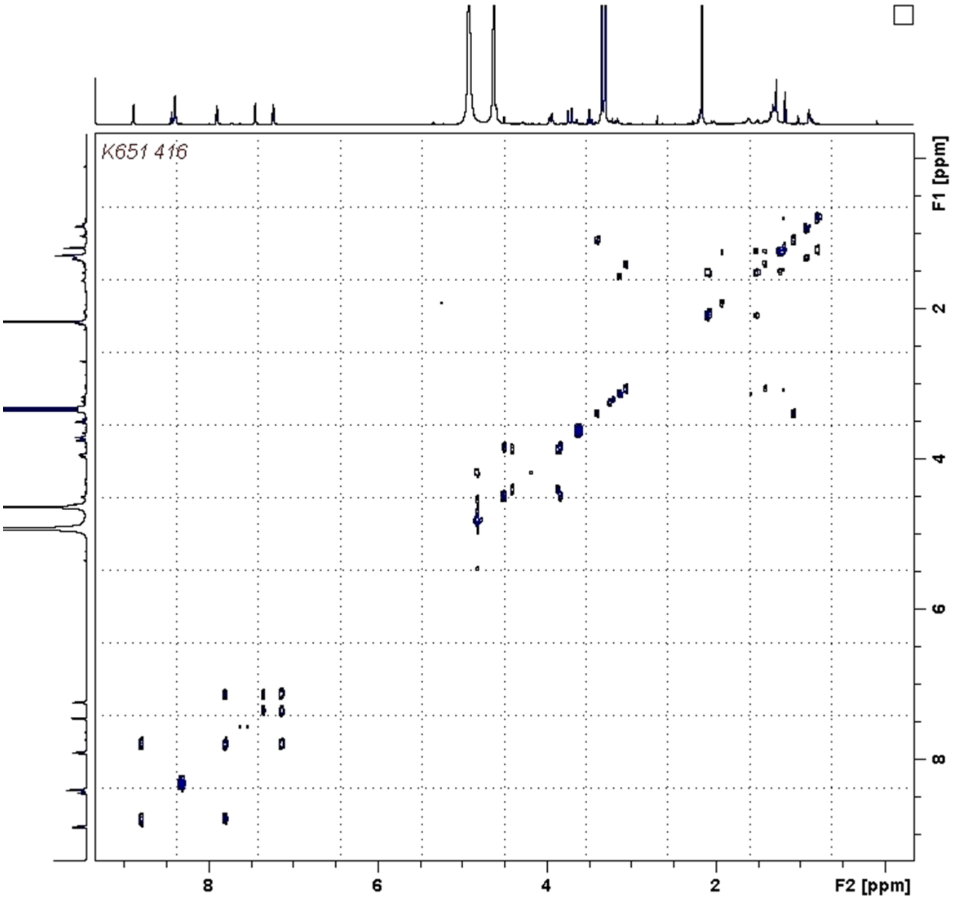

Figure S 2: COSY analysis of $[Os\text{-}(R_4)_2]^{10+}$ conjugate (MeOH-d_4/D_2O, 600 MHz)

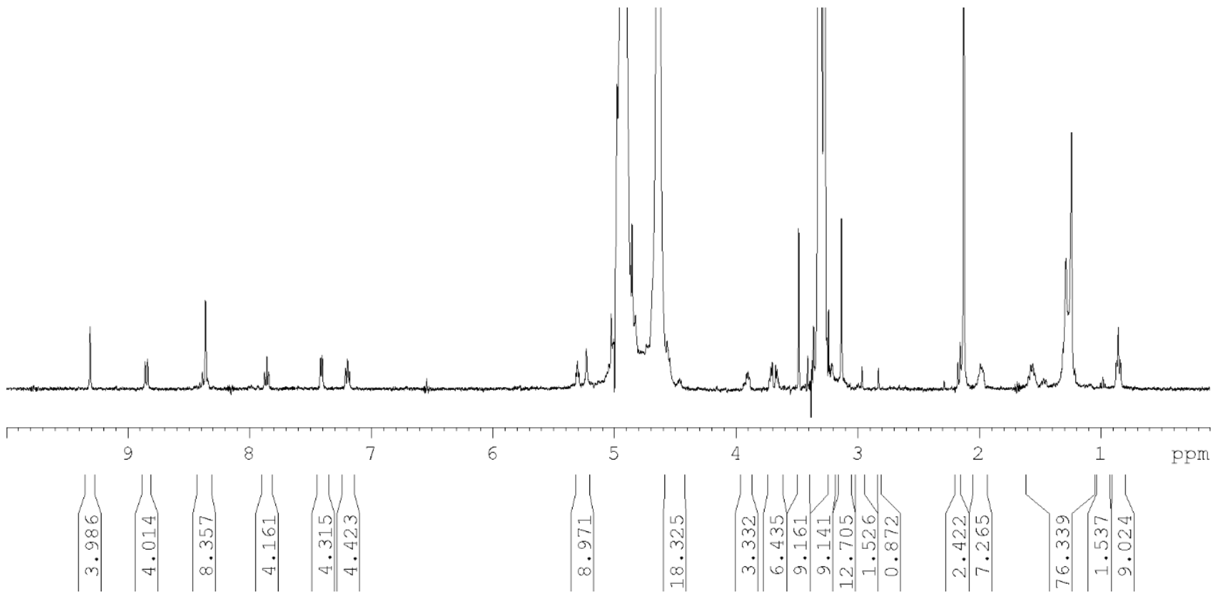

Figure S 3: ^{1}H NMR analysis of $[Os\text{-}(R_8)_2]^{18+}$ conjugate (MeOH-d_4/D_2O, 600 MHz)

Mass Spectrometry

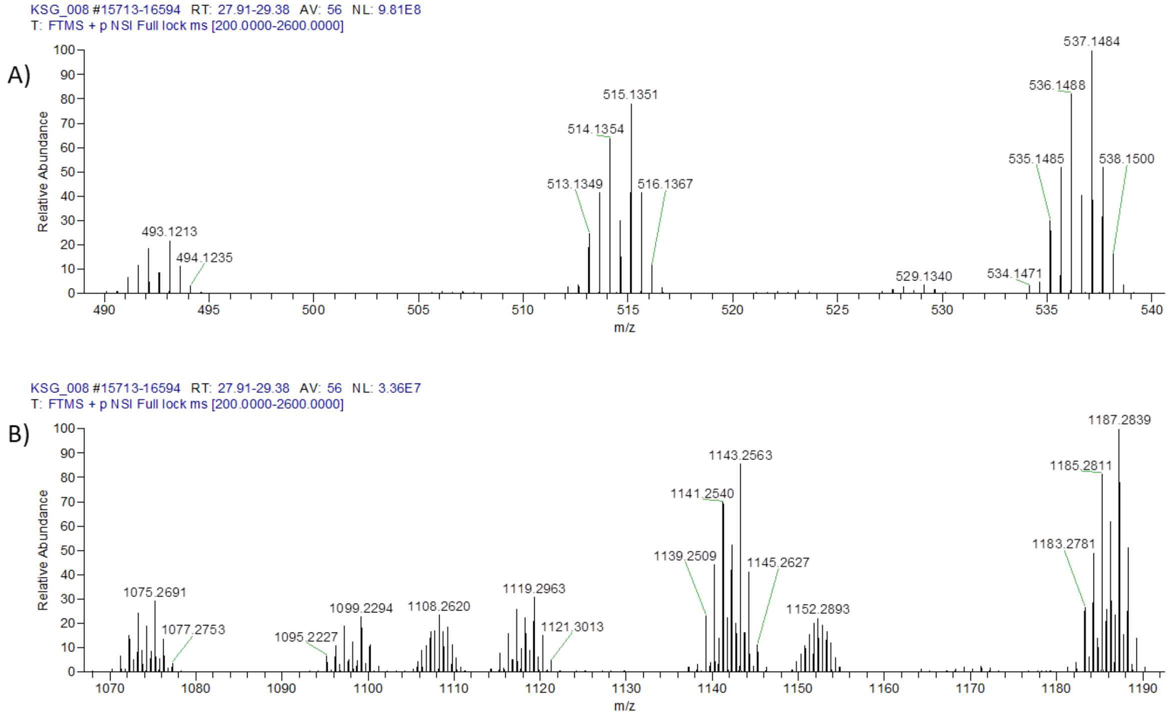

Figure S 4: LC-MS/MS (Q- Exactive) of bis-octaarginine Os(II) conjugate (RT 27.9-29.4 min), zoomed *m/z*, z=2, A) 490 - 540 B) 1070 – 1190.

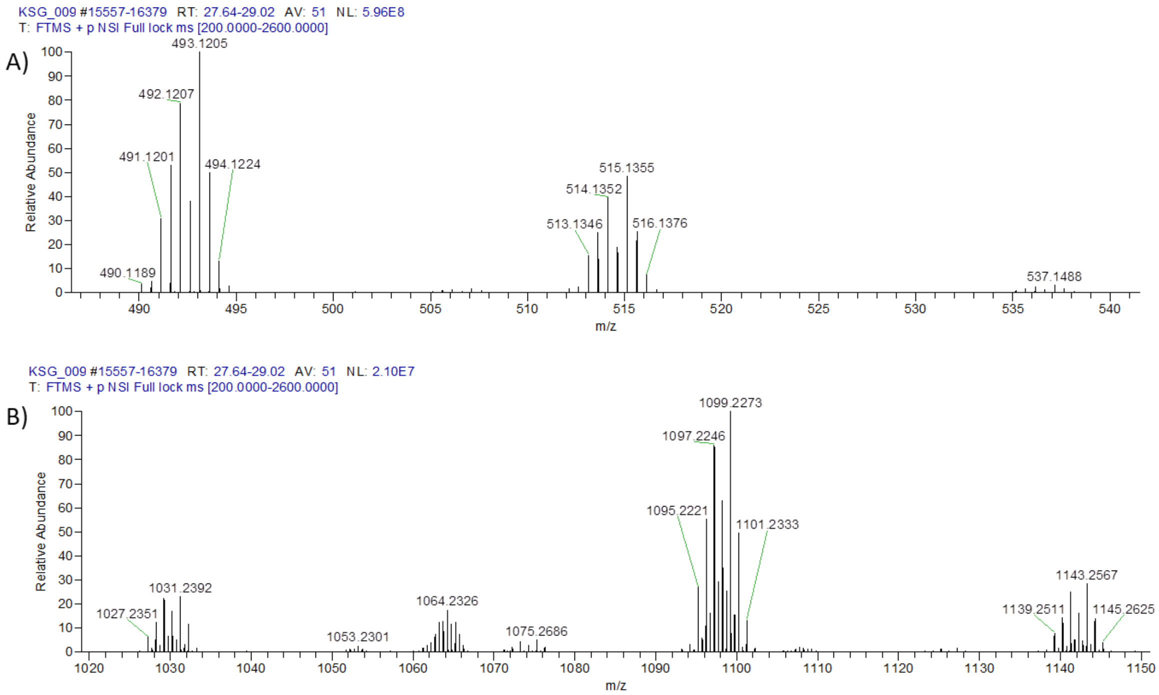

Figure S 5: LC-MS/MS (Q-Exactive) of bis-tetraarginine Os(II) conjugate (RT 27.6 – 29 min), zoomed *m/z*, z=2, A) 490 – 540 and B) 1020-1150.

RP-HPLC Analysis

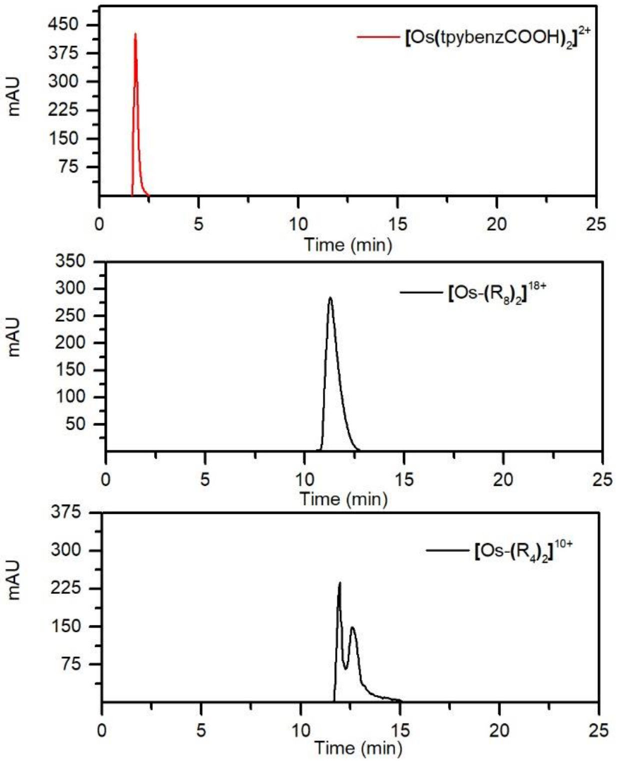

Figure S 6: RP-HPLC analysis of polyarginine Os(II) conjugates relative to $[Os(tpybenzCOOH)_2]^{2+}$ parent complex (C18, $MeCN/H_2O$ (0.1 % TFA) gradient, PDA 490 nm)

Photophysical studies

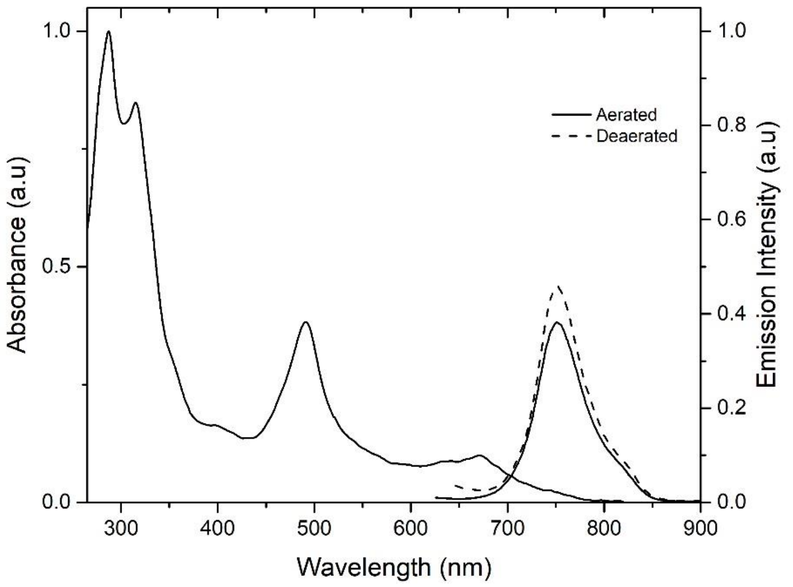

Figure S 7: Absorbance and emission spectra of $[Os\text{-}(R_8)_2]^{18+}$ (Normalised to λ_{max}). Spectra were recorded at 50 µM (PBS buffer pH 7.4/ 0.1 % DMSO) under aerated and deaerated conditions with excitation and emission slit widths of 10 nm and λ_{exc} 490 nm.

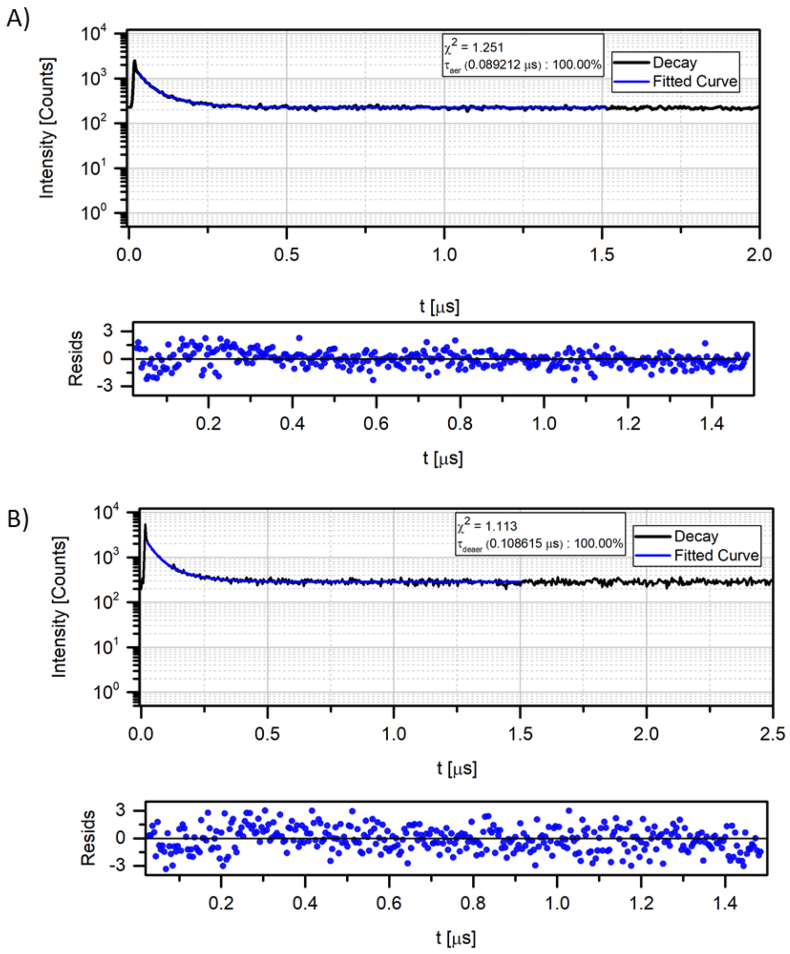

$$\int_{-\infty}^{t} IRF\,(t')\sum_{i-1}^{x} A_i e^{-\frac{t-t'}{t_1}}\,dt'$$

Figure S 8: Emission Decays of bis-octaarginine Os(II) conjugate, $[Os\text{-}(R_8)_2]^{18+}$ (50 µM) in A) aerated and B) deaerated PBS (pH 7.4). Residual plots for the exponential fitting of both curves are shown below each plot.

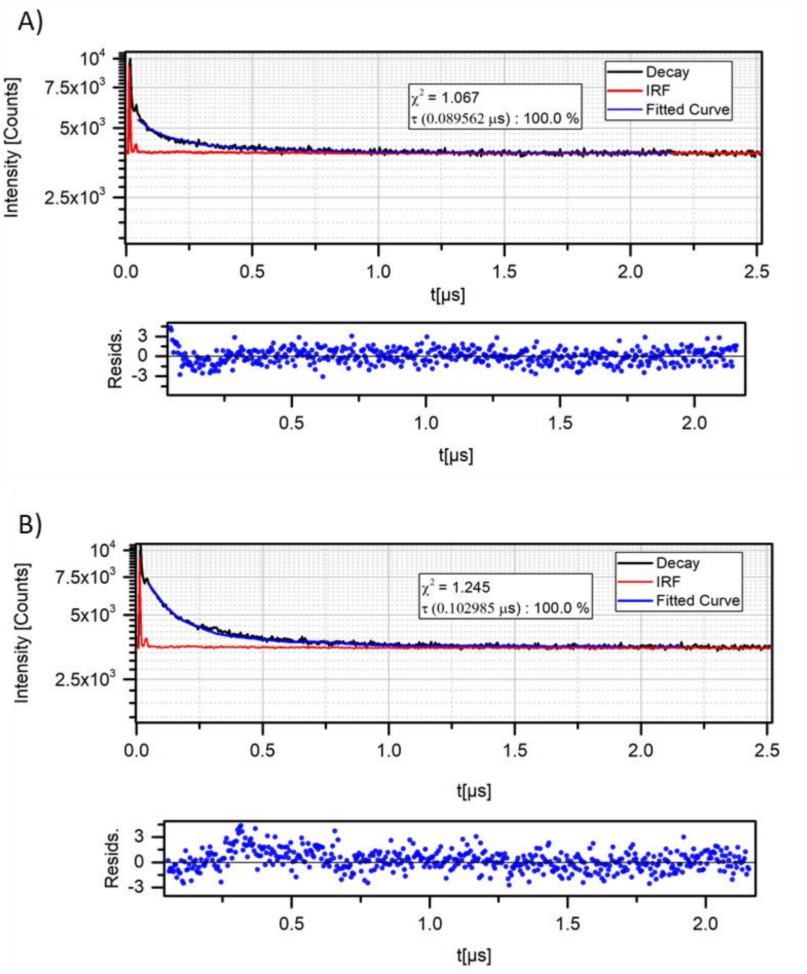

$$\int_{-\infty}^{t} IRF\,(t')\sum_{i-1}^{x} A_i e^{-\frac{t-t'}{t_1}}\,dt'$$

Figure S 9: Emission Decays of bis-tetraarginine Os(II) conjugate, $[Os\text{-}(R_4)_2]^{10+}$ (30 μM) in A) aerated and B) deaerated PBS (pH 7.4). Residual plots for the exponential fitting of both curves are shown below each plot.

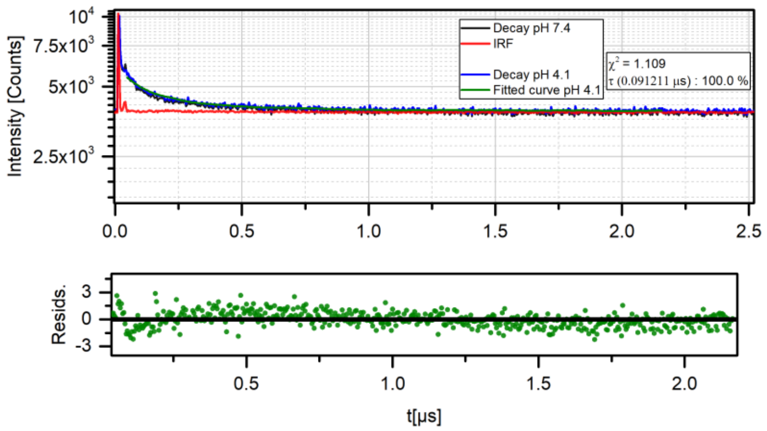

Figure S 10: Emission decay of bis-tetraarginine Os(II) conjugate, $[Os\text{-}(R_4)_2]^{10+}$ (30 μM) in PBS at pH 7.4 and pH 4.1 (using perchloric acid, 1% v/v). The residual plot corresponds to the exponential fitting of the decay at pH 4.1, but the decays and fits are indistinguishable.

Cell studies

Confocal images 2D cell monolayers

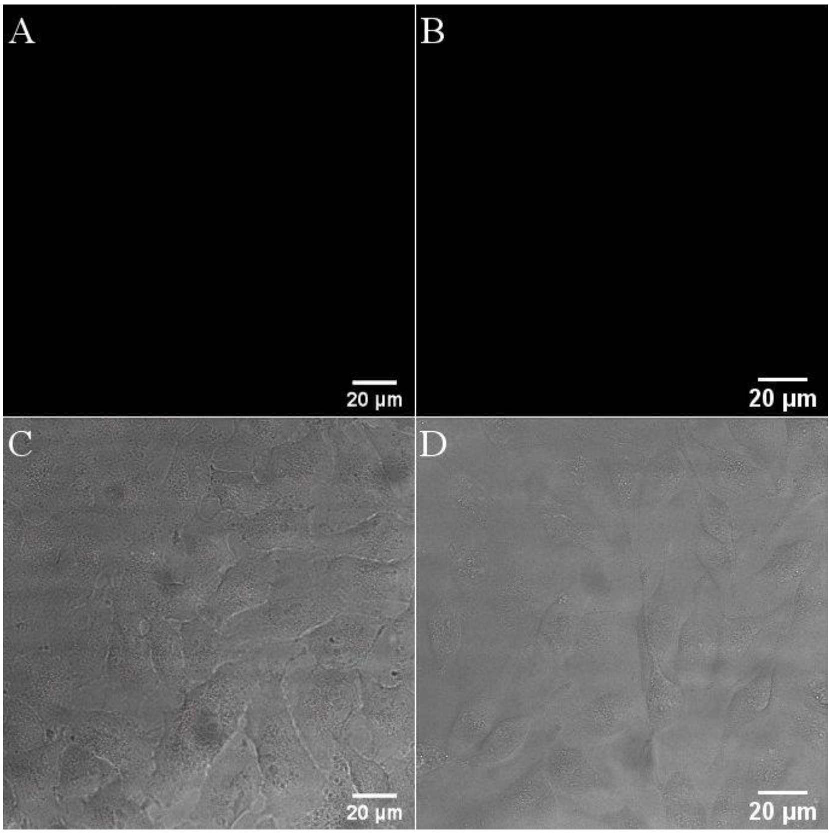

Figure S 11: Confocal images of (A,C) A549 and (B,D) CHO cells treated with parent compound $[Os(tpybenzCOOH)_2]^{2+}$ at 100 µM/ 24 h. Shown are the Os(II) channel and overlay with brightfield. The complex was excited using a 490 nm white light laser and the emission was collected between 650 and 800 nm.

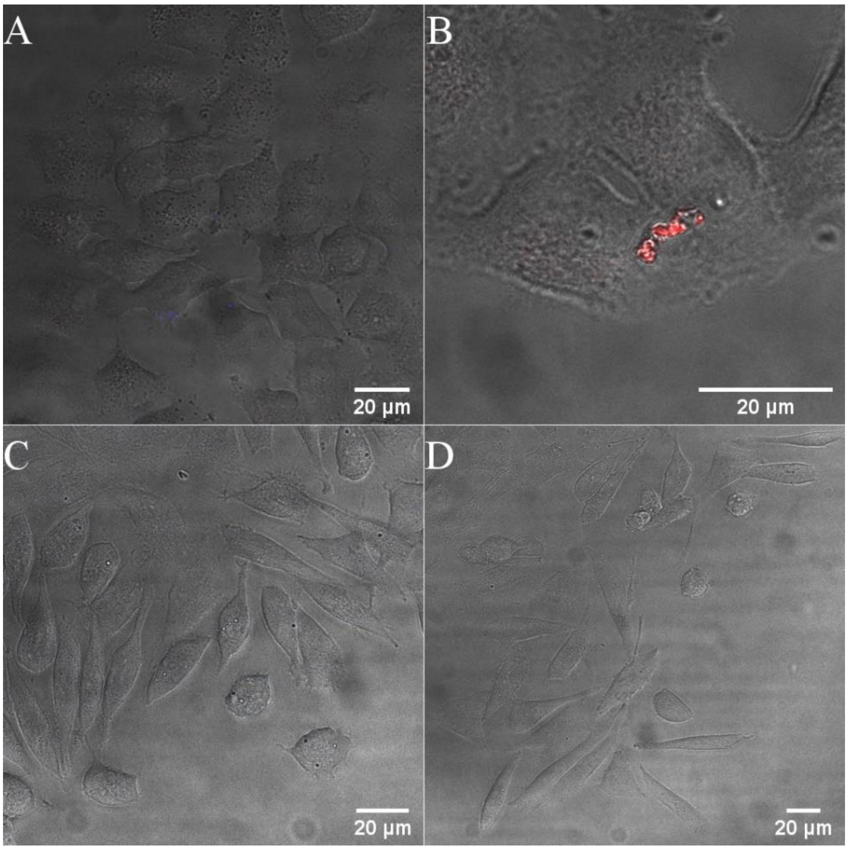

Figure S 12: Confocal images of (A-B) A549 and (C-D) CHO cells treated with $[Os\text{-}(R8)_2]^{18+}$ at 30 µM/ 24 h (left) and 100 µM/ 24 h (right) and co-stained with DRAQ7. Shown are the overlay images of $[Os\text{-}(R8)_2]^{18+}$ and DRAQ7 channel with brightfield. The 633 nm laser was used to excite DRAQ7 and emission was collected between 635–750 nm.

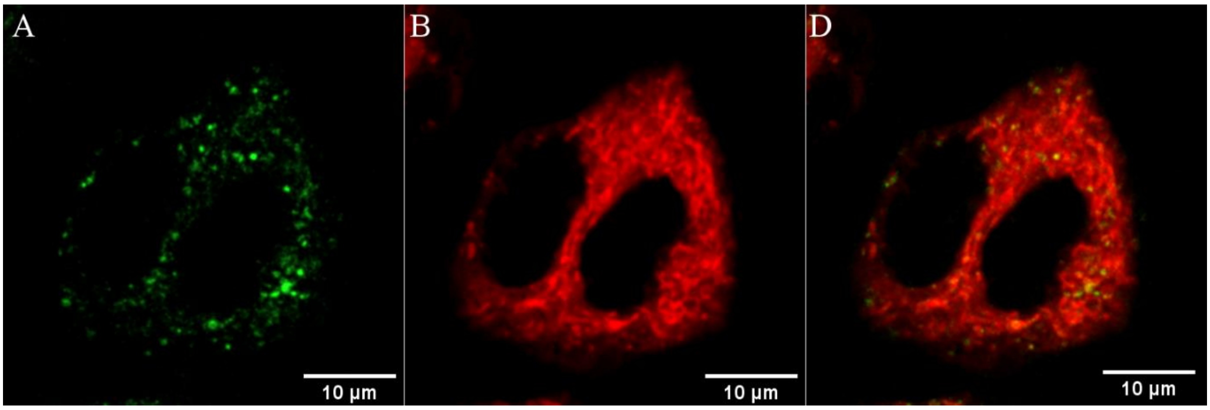

Figure S 13: Confocal fluorescence images of A549 cells treated with (A) $[Os\text{-}(R_4)_2]^{10+}$ (30 µM/ 24 h) and co-stained with (B) MitoTracker Deep Red (100 nM). (C) Overlay of the two channels. The conjugate was excited using a 490 nm white light laser and the emission range was set to between 650 and 800 nm. MitoTracked Deep Red was excited at 644 nm and emission was collected between 655–720 nm

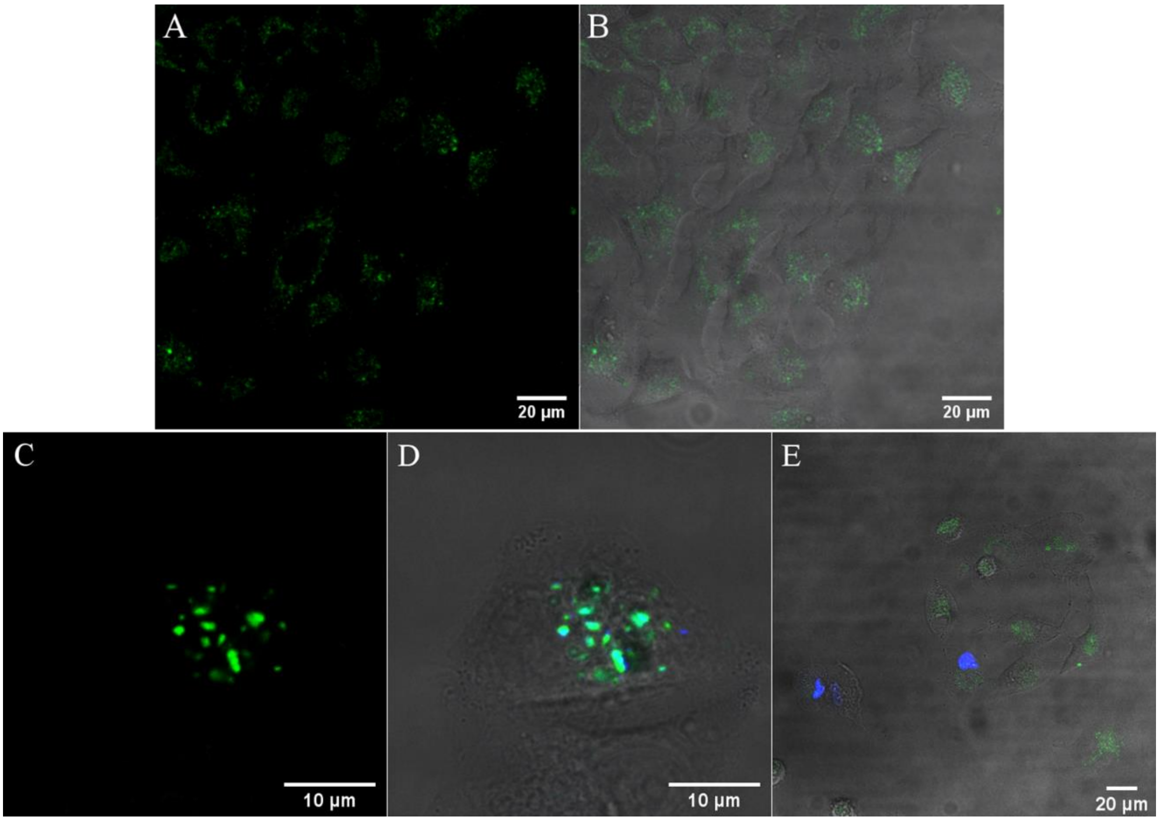

Figure S 14: Representative confocal images of A549 cells treated with 100 µM of $[Os\text{-}(R_4)_2]^{10+}$ and co-stained with DRAQ7 (3 µM). (A) Os(II) channel following 24 h incubation, (B) overlay with DRAQ7 channel and brightfield, (C) Os(II) channel following 48 h incubation, (D) overlay with DRAQ7 channel and brightfield, (E) overlay with DRAQ7 channel and brightfield of a wide region of cells. The 633 nm laser was used to excite DRAQ7 and emission was collected between 635–750 nm.

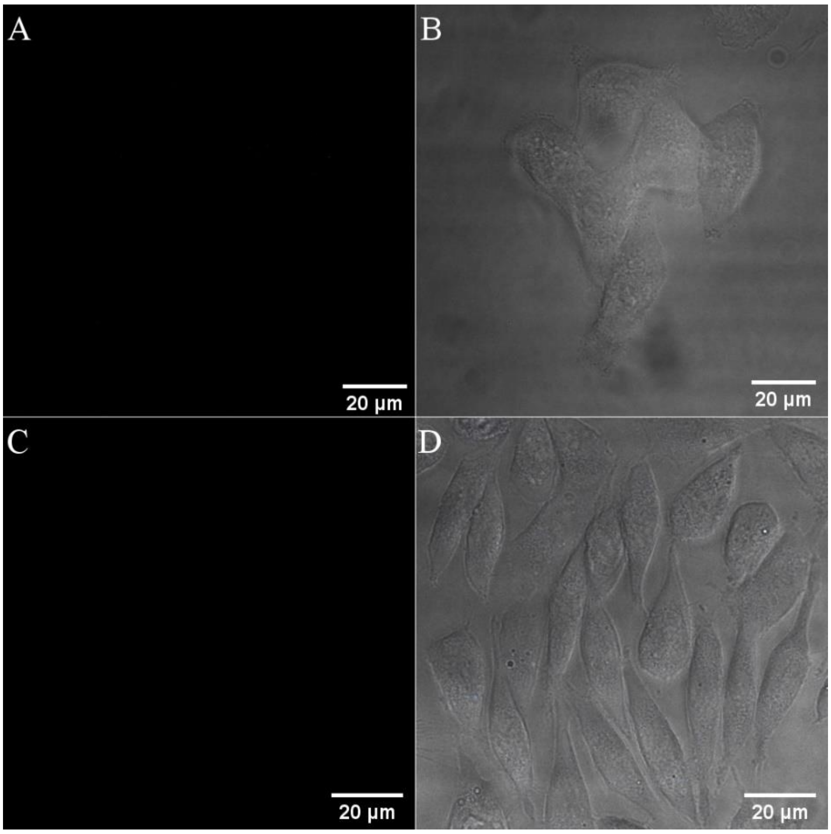

Figure S 15: Confocal images of 4°C uptake studies of $[Os\text{-}(R_4)_2]^{10+}$ at 30 µM/ 24 h where (A-B) A549 and (C-D) CHO cell line. No uptake was observed as shown by the $[Os\text{-}(R_4)_2]^{10+}$ channel (A, C) and overlay with brightfield images. The conjugate was excited using a 490 nm white light laser and the emission range was set to between 650 and 800 nm.

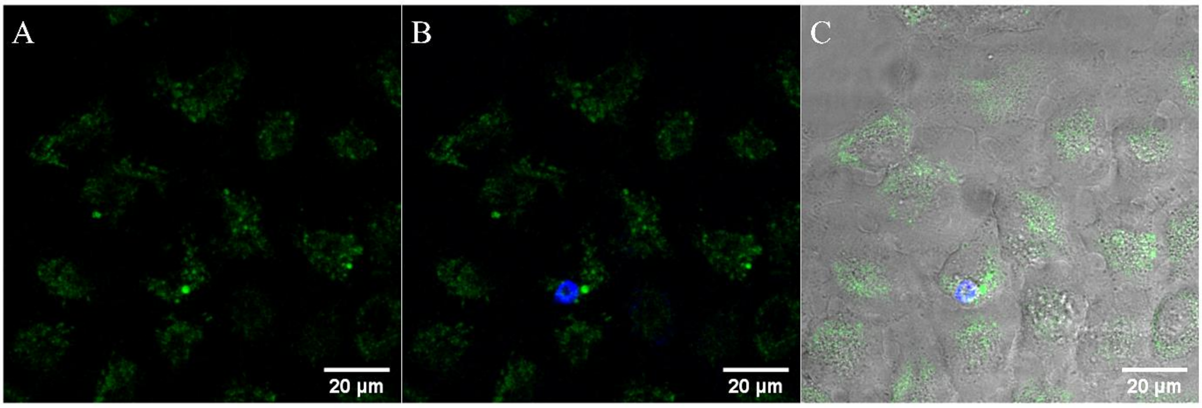

Figure S 16: Representative confocal images of A549 cells treated with 30 µM of $[Os\text{-}(R_4)_2]^{10+}$ for 48 h and co-stained with DRAQ7 (blue). (A) Os(II) channel, (B) Os(II)/DRAQ7 overlay and (C) overlay of channels with brightfield.

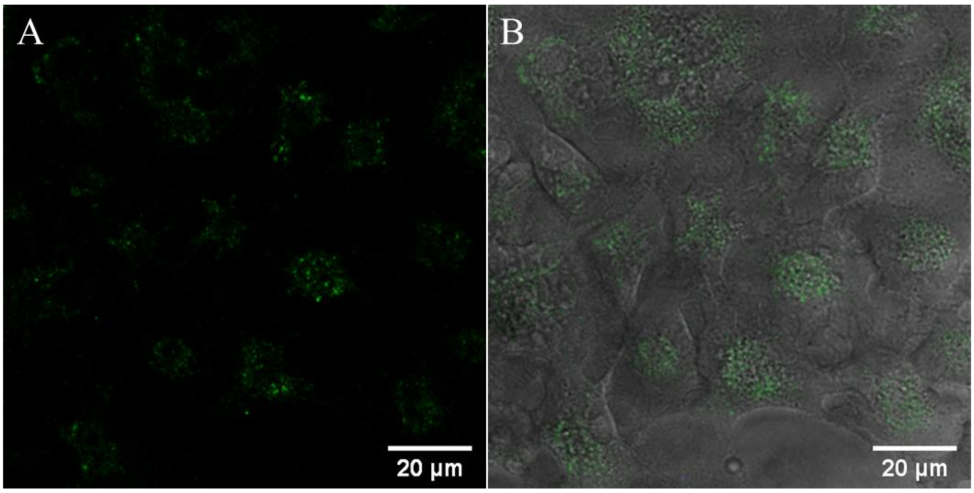

Figure S 17: Confocal images of A549 cells treated with $[Os\text{-}(R_4)_2]^{10+}$ (30 µM/ 24 h) and co-stained with DRAQ7. Shown are the overlay images of the (A) Os(II) channel with DRAQ7 and (B) Os(II)/DRAQ7 with brightfield following continuous irradiation (0.84 µW/cm^2) for 3 h. No photo-induced toxicity was evident as absence of nuclear staining by DRAQ7 (blue) confirmed cell viability

Cytotoxicity assay

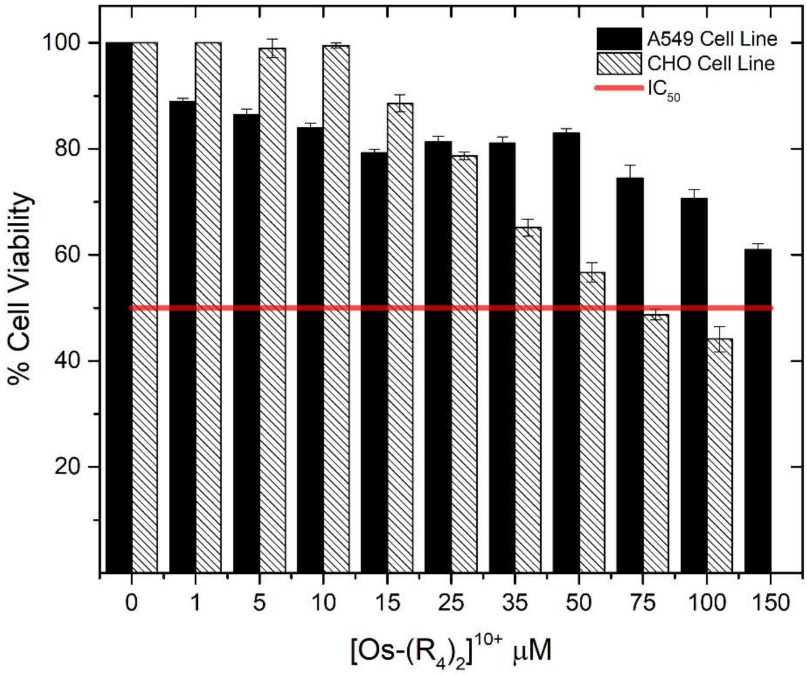

Figure S 18: Cell Viability assay performed for A549 and CHO cells treated with $[Os\text{-}(R_4)_2]^{10+}$ probe at a range of concentrations for 24 h. (n=3)

Luminescence Lifetime Imaging

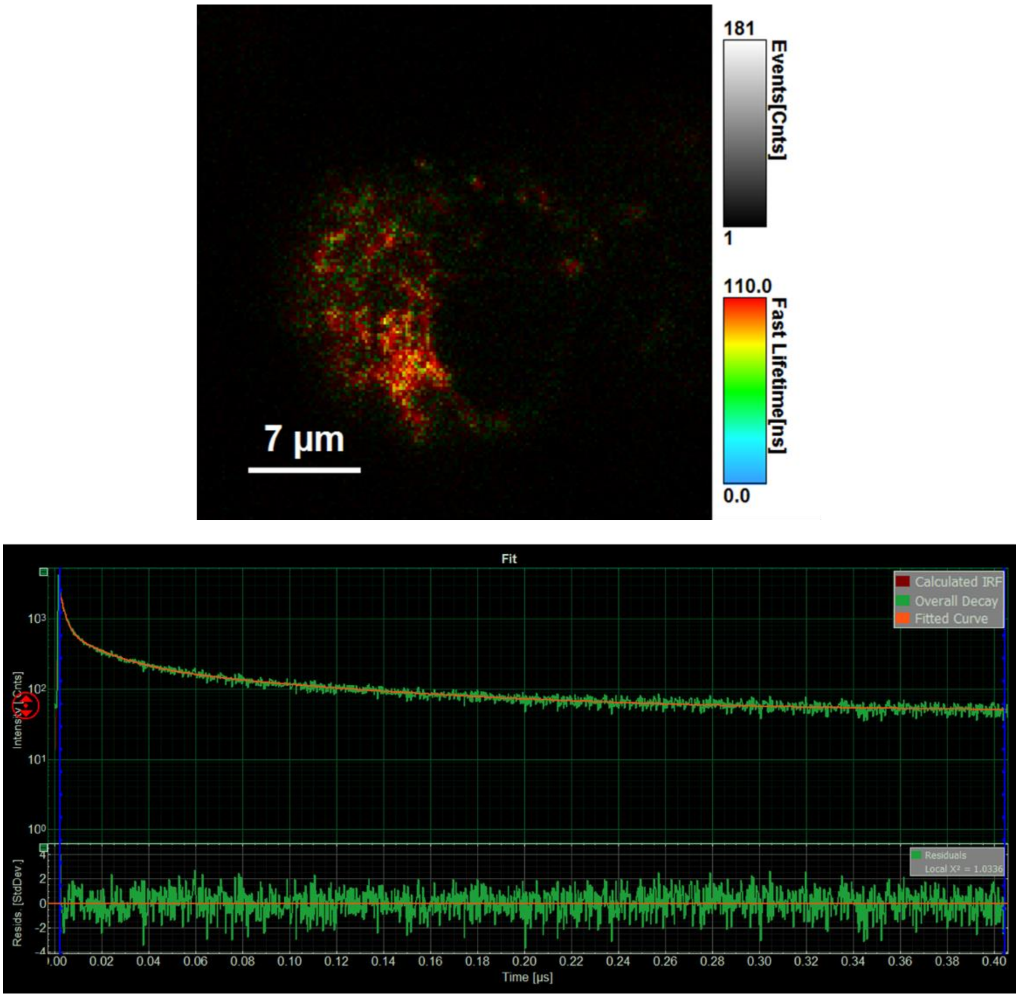

Figure S 19: Representative luminescent lifetime imaging microscopy and fits for $[Os\text{-}(R_4)_2]^{10+}$ (30 μM/ 24 h) in a live A549 cell. The decay was fitted to a tri-exponential model with lifetimes of 92.2 ns (67 %), 15.8 ns (25 %) and 2.02 ns (8 %) (χ^2 = 1.0336). The PLIM image was acquired by exciting at 405 nm and emission collected between 650 and 800nm.

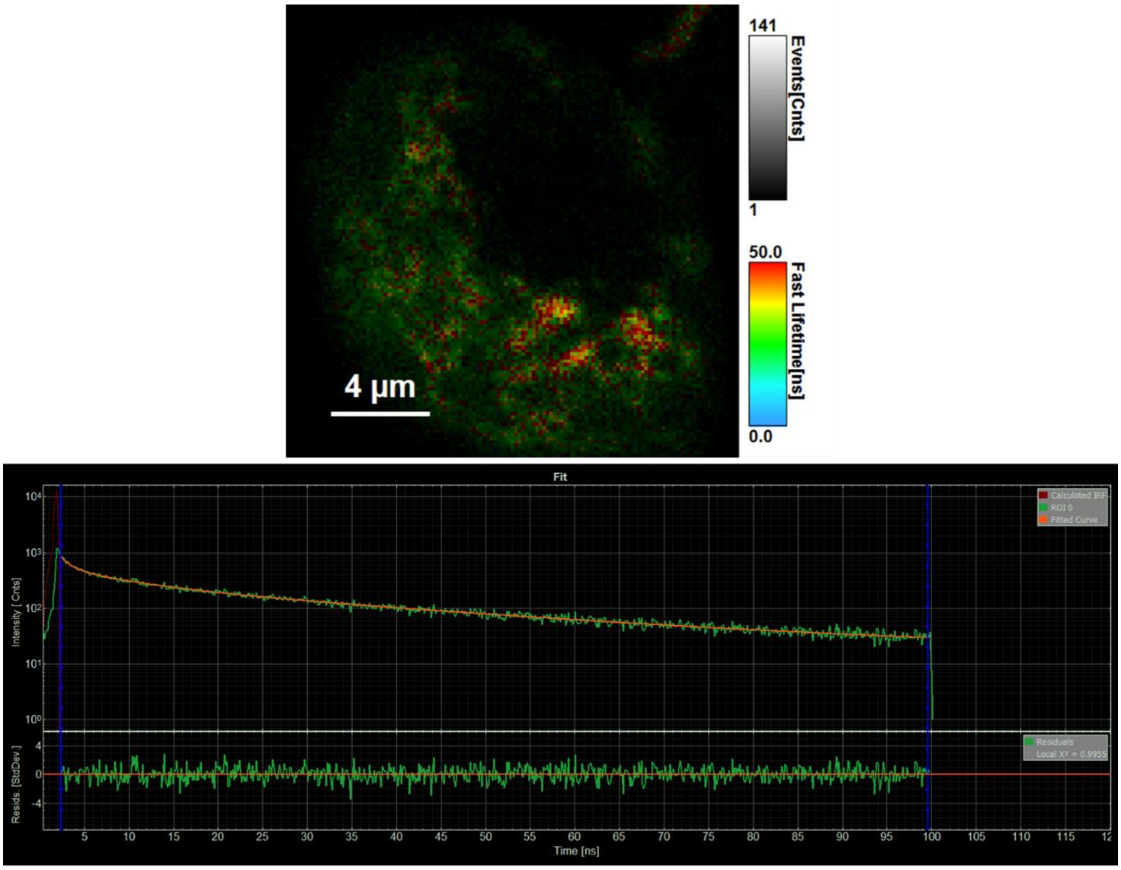

Figure S 20: Representative luminescent lifetime imaging microscopy of $[Os\text{-}(R_4)_2]^{10+}$ (30 µM/ 48 h) in a live A549 cell. The decay was fitted to a tri-exponential model with lifetimes of 37.0 ns (54 %), 9.3 ns (32 %) and 1.88 ns (14 %). (χ^2 = 0.9955). The PLIM image was acquired by exciting at 405 nm and emission collected between 650 and 800nm.

3D Multicellular pancreatic spheroids

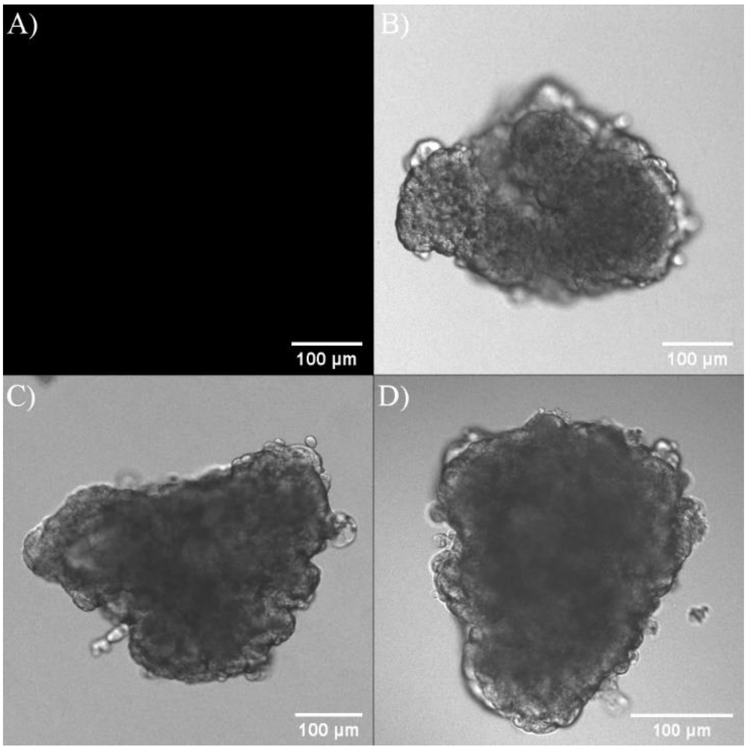

Figure S 21: Confocal images of representative live HPAC spheroid control samples. (A) Os(II) channel of non-treated control spheroid sample, (B) overlay of channel with brightfield, (C) Os(II)/Brightfield overlay of spheroid incubated with parent complex $[Os(tpybenzCOOH)_2]^{2+}$ at 100 µM/ 48 h (D) Os(II)/Brightfield overlay of spheroid incubated with bis-octaarginine $[Os\text{-}(R_8)_2]^{18+}$ at 100 µM/ 48 h. A 490 nm white light laser was used for excitation and emission for Os(II) was set to 650 – 800 nm.

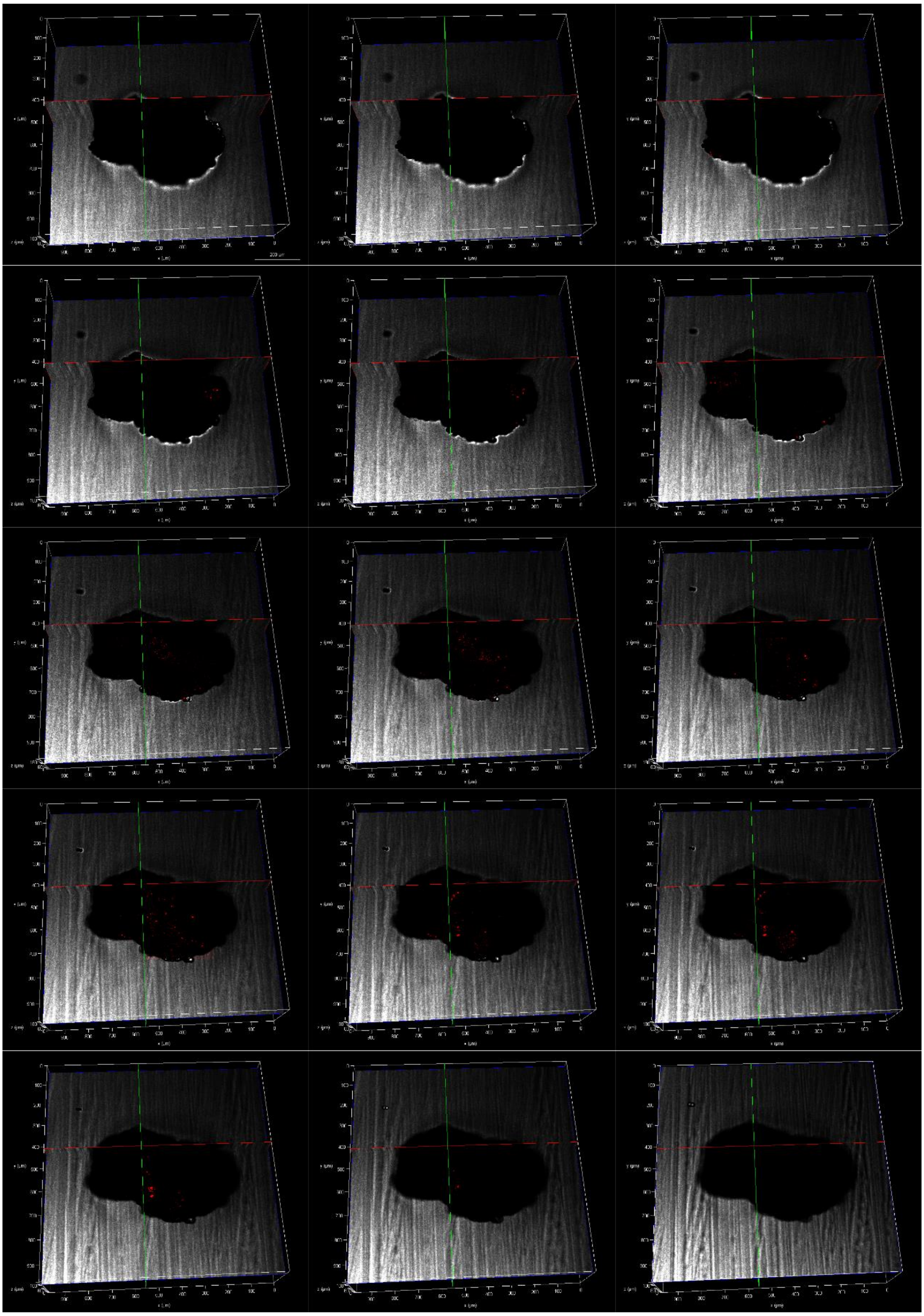

Figure S 22: Z-stack images of a single live HPAC spheroid pre-treated with $[Os\text{-}(R_4)_2]^{10+}$ for at 100 μM/ 24 h. Each image corresponds to cross-section from the bottom (upper left image) to the upper part lower right image) along the z-axis. Scale bar reads 200 μm. Os(II) was excited at 490 nm and emission was collected between 650 and 850 nm. Representative slices are shown using contrast BF as the background .(10X)

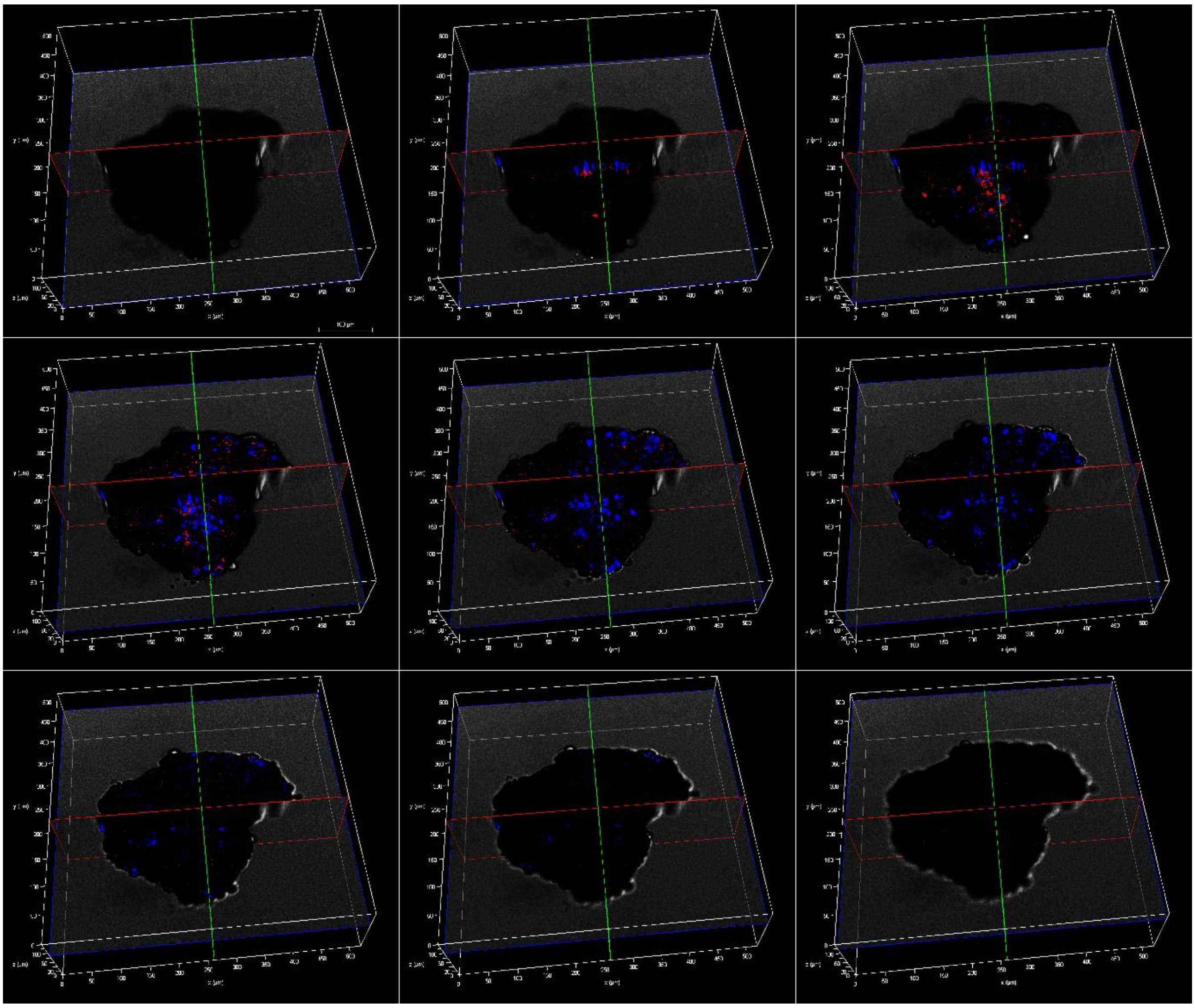

Figure S 23: Z-stack images of a single live HPAC spheroid pre-treated with Os-$(R_4)_2$ (100 µM/ 24 h) and co-stained with DAPI. Each image corresponds to cross-section from the bottom (upper left image) to the upper part (lower right image) along the z-axis. Os(II) was excited at 490 nm and emission was collected between 650 and 850 nm. DAPI was excited at 405 nm and emission was collected between 423 nm and 580 nm. Representative images are shown using contrast brightfield as the background . Scale bar reads 100 µm. (10X)

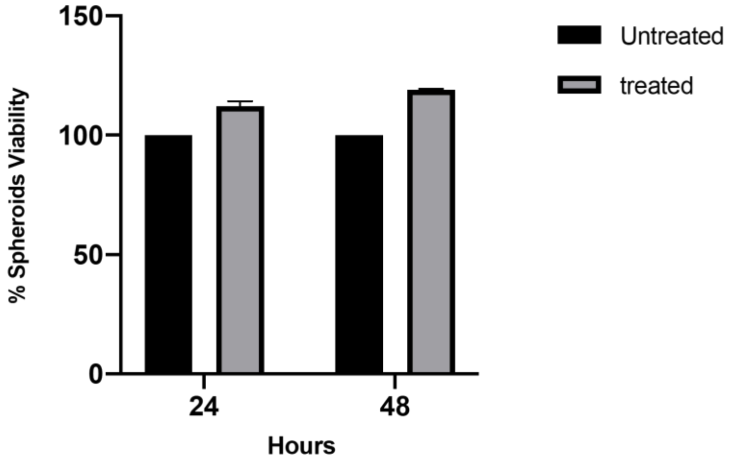

Figure S 24: HPAC spheroid viability was measured after 24 and 48 hrs. of incubation with $[Os\text{-}(R_4)_2]^{10+}$ at 100 μM. (n=2)

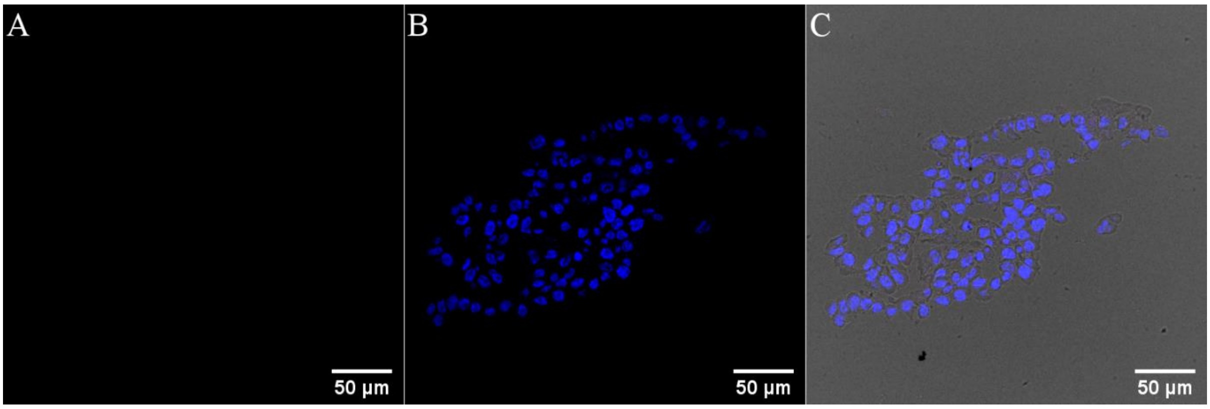

Figure S 25: Fixed slices of control HPAC spheroids treated only with DAPI dye. (A) Os (II) channel, (B) DAPI emission was collected between 423 nm and 580 nm. (C) Overlay with brightfield.

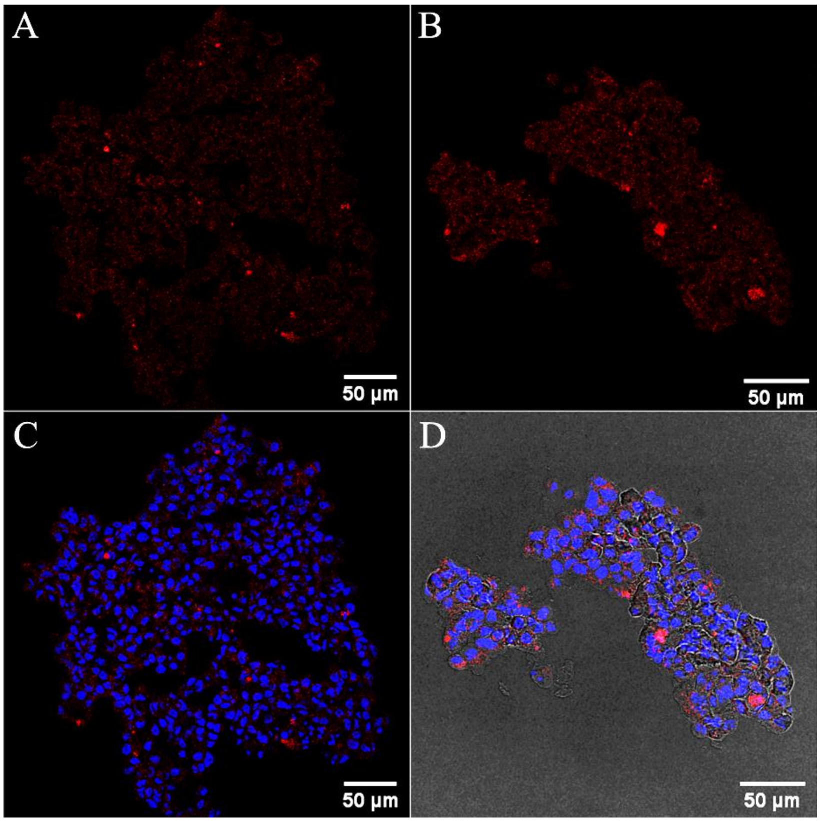

Figure S 26: Fixed cryosections of HPAC spheroids pre-treated with $[Os\text{-}(R_4)_2]^{10+}$ at 100 µM / 24 h and co-stained with DAPI post-fixation. (A-B) Os(II) emission channel from two representative cryosections, (C) co-staining with DAPI and (D) Os(II)/DAPI overlay with brightfield. A 490 nm white light laser was used to excite the conjugate and emission was collected between 650 and 800 nm.. The 405 nm excitation laser was used to excite DAPI and emission was collected between 423 and 580 nm.

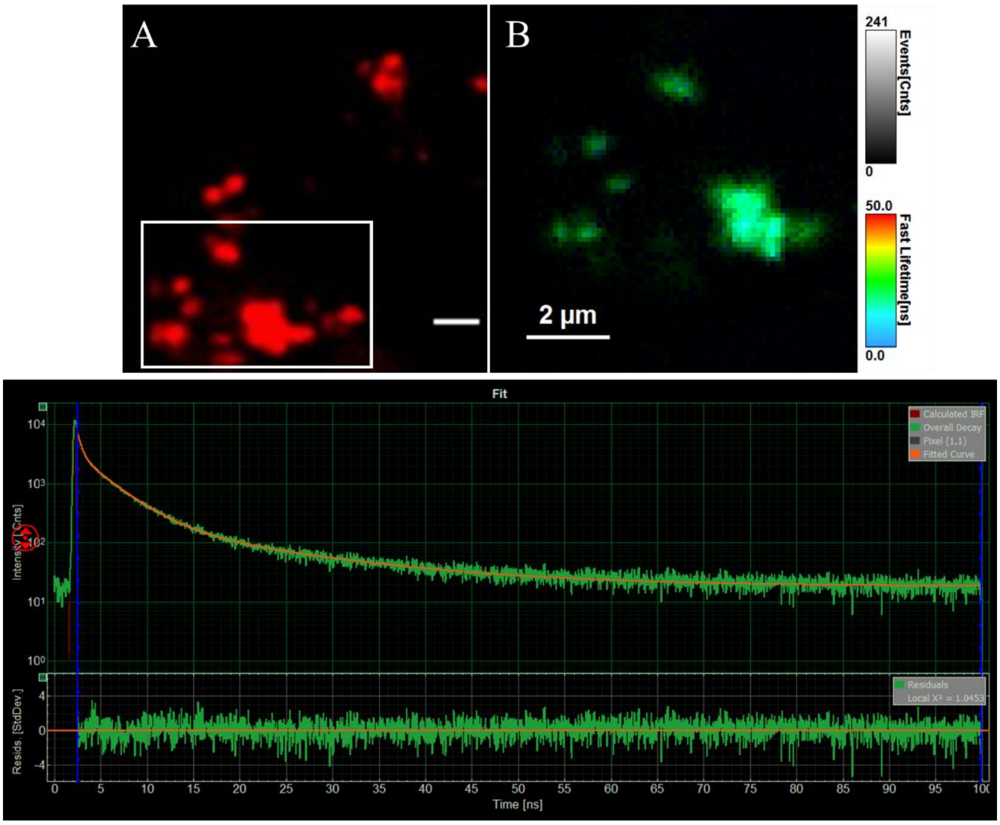

Figure S 27: A) Confocal and B) Luminescence lifetime imaging of $[Os\text{-}(R_4)_2]^{10+}$ in fixed HPAC spheroid slice. The PLIM decay was fitted to a bi-exponential model with lifetimes of 16.4 ns, and 3.2 ns (χ^2 = 1.0453). The PLIM image was acquired by exciting at 405 nm and emission collected between 650 and 800nm.
